# Newly Discovered Occurrences and Gene Tree of the Extracellular Globins and Linker Chains from the Giant Hexagonal Bilayer Hemoglobin in Metazoans

**DOI:** 10.1093/gbe/evz012

**Published:** 2019-01-21

**Authors:** Flávia A Belato, Carlos G Schrago, Christopher J Coates, Kenneth M Halanych, Elisa M Costa-Paiva

**Affiliations:** 1 Laboratório de Biologia Evolutiva Teórica e Aplicada, Departamento de Genética, Universidade Federal do Rio de Janeiro, RJ, Brazil; 2 Department of Biosciences, College of Science, Swansea University, United Kingdom; 3 Department of Biological Sciences, Molette Biology Laboratory for Environmental and Climate Change Studies, Auburn University; 4 Departamento de Zoologia, Instituto Biociências, Universidade de São Paulo, SP, Brazil

**Keywords:** chlorocruorins, erythrocruorins, extracellular hemoglobins, transcriptome, oxygen transport, gene tree

## Abstract

Multicellular organisms depend on oxygen-carrying proteins to transport oxygen throughout the body; therefore, proteins such as hemoglobins (Hbs), hemocyanins, and hemerythrins are essential for maintenance of tissues and cellular respiration. Vertebrate Hbs are among the most extensively studied proteins; however, much less is known about invertebrate Hbs. Recent studies of hemocyanins and hemerythrins have demonstrated that they have much wider distributions than previously thought, suggesting that oxygen-binding protein diversity is underestimated across metazoans. Hexagonal bilayer hemoglobin (HBL-Hb), a blood pigment found exclusively in annelids, is a polymer comprised up to 144 extracellular globins and 36 linker chains. To further understand the evolutionary history of this protein complex, we explored the diversity of linkers and extracellular globins from HBL-Hbs using in silico approaches on 319 metazoan and one choanoflagellate transcriptomes. We found 559 extracellular globin and 414 linker genes transcribed in 171 species from ten animal phyla with new records in Echinodermata, Hemichordata, Brachiopoda, Mollusca, Nemertea, Bryozoa, Phoronida, Platyhelminthes, and Priapulida. Contrary to previous suggestions that linkers and extracellular globins emerged in the annelid ancestor, our findings indicate that they have putatively emerged before the protostome–deuterostome split. For the first time, we unveiled the comprehensive evolutionary history of metazoan HBL-Hb components, which consists of multiple episodes of gene gains and losses. Moreover, because our study design surveyed linkers and extracellular globins independently, we were able to cross-validate our results, significantly reducing the rate of false positives. We confirmed that the distribution of HBL-Hb components has until now been underestimated among animals.

## Introduction

Aerobic cells require oxygen for their maintenance and growth; therefore, multicellular organisms such as metazoans depend on proteins for transporting oxygen from the external environment to body tissues (Terwilliger 1998). These proteins play an essential role in organismal homeostasis, and hence have been extensively investigated (Riggs 1976). Hemoglobins (Hbs) occur in all domains of life displaying an extraordinary diversity of form and function, among which vertebrate Hbs stand out as the most studied blood pigments of all (Royer 1992; Vinogradov et al. 2006, 2007; Gell 2018). Even though invertebrate Hbs present a wide variation of structures, much less is known about their diversity, distribution, and evolutionary history. Recent genomic and transcriptomic studies demonstrated that the known diversity of oxygen-carrying proteins in animals, such as hemerythrins (Hrs) and hemocyanins (Hcs), was underestimated (Bailly et al. 2008; Martín-Durán et al. 2013; Costa-Paiva et al. 2017, 2018). For instance, Hrs were previously thought to be present only in four invertebrate phyla (Annelida, Brachiopoda, Priapulida, and Bryozoa) (Kurtz 1992), and that Hcs were exclusive to Mollusca and Arthropoda (Burmester 2002). However, Bailly et al. (2008) reported a broad distribution of Hrs among annelids. Subsequently, Martín-Durán et al. (2013) and Costa-Paiva et al. (2017, 2018) discovered Hcs and Hrs among other metazoans, with novel records in seven and nine animal phyla, respectively. These data consistently showed the extent of the underestimation of oxygen transport/binding protein diversity in metazoans.

**Fig. 1.**
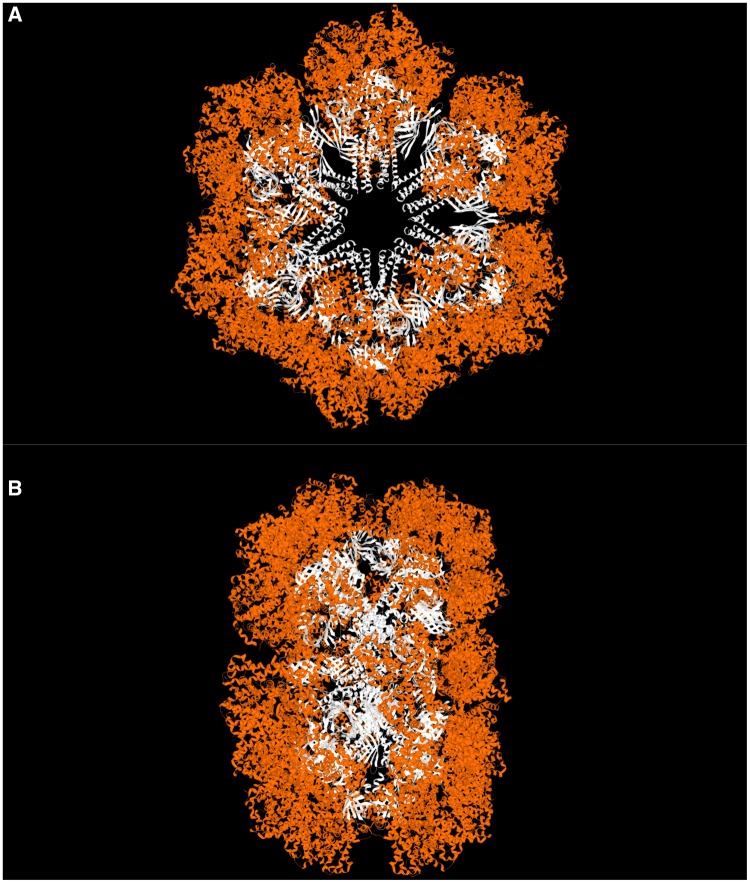
—*Lumbricus terrestris* hexagonal bilayer hemoglobin cryo-EM complex (EMD-2627/PDB 4V93). (*A*) Front view of the whole molecule. (*B*) Top view of the whole molecule. Extracellular globin subunits, at the outside of the molecule, are shown in orange. Linker subunits, in the interior of the molecule, are shown in white.

The giant, extracellular, hexagonal bilayer hemoglobin (HBL-Hb) is a protein complex involved in oxygen transport in annelids (Vinogradov 1985; Weber and Vinogradov 2001). This protein group presents a unique quaternary structure and is known in a variety of annelids (Zal and Rousselot 2014). HBL-Hbs have high molecular masses in the range of 3,000–4,000 kDa and comprise 180 polypeptides chains, which are grouped into two categories: globins and nonglobins (Lamy et al. 1996). The structure of earthworm (*Lumbricus terrestris*) HBL-Hb, which is among the best studied, contains 144 globin chains (each of which binds O_2_ reversibly), arranged into 12 dodecamers that assemble around a central core of 36 linker chains (12 trimers) (fig. 1) (Lamy et al. 2000; Royer et al. 2005, 2006). HBL-Hbs are synthesized intracellularly and secreted. Their extracellular location in blood vascular or coelomic fluid systems seems to correlate with their large molecular size, and prevents their excretory loss through tissue membranes (Weber and Vinogradov 2001). The protein complex is extremely stable, being resistant to autoxidation, and is capable of transporting O_2_ to tissues when transfused into mammals without producing side effects (Harrington et al. 2007). Such features make HBL-Hb a promising candidate to act as a blood substitute in human transfusions/therapeutics and for the preservation of organs, tissues, and cells (Zal and Rousselot 2014; Zal et al. 2014). HBL-Hb represents one of four groups of invertebrates extracellular Hbs, which comprised a multisubunit complex made of globins with a single oxygen-binding site in each one of them, and linker chains that are devoid of heme (Vinogradov 1985; Weber and Vinogradov 2001). Giant extracellular Hbs were also referred to as erythrocruorins or chlorocruorins, but there was a lack of uniformity in usage of these terms. Therefore, those names are no longer employed.

The pioneering work of Gotoh et al. (1987) divided HBL-Hb globins into two main chains, A and B, based on their primary structures. Later, Yuasa et al. (1996) corroborated these results and suggested a further division into four paralogous globin types: A1/A2 and B1/B2. According to them, the gene duplication events that originated the four subtypes occurred before the Annelida diversification. Suzuki et al. (1990) was the first to describe the two linker chains, L1 and L2, based on their sequences. Suzuki and Riggs (1993) described one additional L3 chain and suggested that it was the result of an ancestral globin gene duplication event and sets of paralogous genes. They also demonstrated that linkers have a conserved cysteine-rich domain that is not present in globin sequences, but homologous to the low-density lipoprotein receptor class A (LDL-A) found in many metazoans, for example, humans and frogs (Sudhof et al. 1985; Mehta et al. 1991). The LDL-A module makes linkers conformationally capable of binding the globin dodecamers to form the hexagonal bilayer structure of the HBL-Hb complex (Suzuki and Riggs 1993). Fushitani et al. (1996) described a fourth linker chain L4; however, this chain appears to be a minor component, most likely a variation of the L3 chain (Vinogradov 2004; Royer et al. 2006). Nevertheless, the evolutionary history of linkers remains unclear.

Previous studies concerning the evolutionary history of extracellular globins and linker chains have been conducted on a very limited sampling of annelid species. Given the recent discoveries of additional Hcs and Hrs with expanded sampling (Bailly et al. 2008; Martín-Durán et al. 2013; Costa-Paiva et al. 2017, 2018), we wanted to revise the evolution of Hbs and in particular HBL-Hbs. Therefore, to better understand the diversity and expression of extracellular globins and linkers that are used in making HBL-Hbs, we interrogated transcriptomic data from across bilaterians, including heavy sampling of annelids. Consistently with other oxygen-carrying proteins, namely hemocyanin and hemerythrin, our results confirm a much broader distribution of extracellular globins and linkers across metazoan, suggesting that the underestimation of oxygen-carrying proteins across animals is a pattern.

## Materials and Methods

Transcriptomes of 319 metazoans from 16 phyla and one choanoflagellate were employed in this work, and information about all species is listed in supplementary file 1, Supplementary Material online. Transcriptomic data were originally obtained as part of the WormNet II project to resolve annelid phylogeny. Samples were collected using several techniques, including intertidal sampling, dredge, and box cores. All samples were preserved in RNALater or frozen at −80 °C. RNA extractions, cDNA preparation, and sequencing followed Kocot et al. (2011) and Whelan et al. (2015). Total RNA was extracted either from whole animals or from the body wall. After extraction, RNAs were purified using TRIzol (Invitrogen) or the RNeasy kit (Qiagen) with on-column DNase digestion. The SMART cDNA Library Construction Kit (Clontech) was used to reverse transcribe single-stranded RNA template. Double-stranded cDNA synthesis was completed with the Advantage 2 PCR system (Clontech). The Genomic Services Lab at the Hudson Alpha Institute (Huntsville, AL) barcoded and sequenced libraries with Illumina technology. Paired-end runs were 100 or 125 bp in length, utilizing either v3 or v4 chemistry on Illumina HiSeq 2000 or 2500 platforms (San Diego, CA). Finally, paired-end transcriptome data were digitally normalized to an average k-mer coverage of 30 using normalize-by-median.py (Brown et al. 2012) and assembled using Trinity r2013-02-25 with default settings (Grabherr et al. 2011).

To search in silico for putative extracellular globin and linker genes associated with HBL-Hbs, we employed the Trinotate annotation pipeline (http://trinotate.github.io/; last accessed December 11, 2018) (supplementary file 2, Supplementary Material online) (Grabherr et al. 2011), which uses a BLAST-based method against the EggNOG 4.5.1 (Huerta-Cepas et al. 2016) and KEGG (Kanehisa et al. 2016) databases to provide the Gene Ontology (GO) annotation. The GO is a standardized functional classification system for genes that describes the properties of genes and their products using a dynamic-updated controlled vocabulary (Gene Ontology Consortium 2004). The Trinotate pipeline uses the following software: HMMER 3.2.1, for protein domain identification (Finn et al. 2011); tmHMM 2.0, for prediction of transmembrane helices in proteins (Krogh et al. 2001); RNAmmer 1.2, for prediction of ribosomal RNA (Lagesen et al. 2007); SignalP 4.1, to predict signal peptide cleavage sites (Petersen et al. 2011); GOseq, for prediction of the GO (Young et al. 2010); and EggNOG 4.5.1, for searching orthologous groups (Huerta-Cepas et al. 2016). Because HBL-Hb comprised two major protein components—linkers and globins—two independent searches were performed in attempt to provide a cross-validation of the results of each one of the searches, aiming to eliminate false-positive records. Retrieved sequences were manually verified by inspecting each functional annotation in order to select sequences annotated with Trinotate as linkers or extracellular globins. In one search, sequences annotated as linkers were retained, whereas extracellular globins were retrieved from a separated search. Contigs identified putatively as linkers or extracellular globins were subsequently translated into amino acids using TransDecoder with default settings (https://transdecoder.github.io/; last accessed October 3, 2018). TransDecoder may produce multiple open reading frames; therefore, all translated amino acid sequences were evaluated through the Pfam domain check (Finn et al. 2016) using the EMBL-EBI protein database with an *e*-value cutoff of 10^−10^. Sequences with a confirmed linker or extracellular globin Pfam domain, and with more than 200 and 130 amino acids respectively, were analyzed further. Manual evaluation was performed in order to verify the presence of the characteristic cysteine-rich amino acid signature found in linkers, including LDL-A: Cys-X_(5__–__7)_-Cys-X_(5__–__6)_-Cys-X_(6)_-Cys-Asp-X_(3)_-Asp-Cys-X_(4)_-Asp-Glu-X_(2__–__4)_-Cys (supplementary file 3, Supplementary Material online) (Negrisolo et al. 2001; Chabasse, Bailly, Sanchez, et al. 2006). For extracellular globins, the 12 invariant amino acid residues found in the main chains A and B (Cys-19, Trp-31, Phe-63, Val-66, Phe-76, His-79, Arg-82, His-106, Gln-110, His-111, Trp-144, and Cys-147) were used to confirm identity, including the Cys-19 residue which is the main feature that differs the extracellular globins from the intracellular ones (supplementary file 4, Supplementary Material online) (Shishikura et al. 1986; Gotoh et al. 1987; Yuasa et al. 1996; Negrisolo et al. 2001; Chabasse, Bailly, Rousselot, et al. 2006). Sequences retained after all those steps were considered as linkers or extracellular globin genes and thus were separated into two respective protein data sets.

**Fig. 2.**
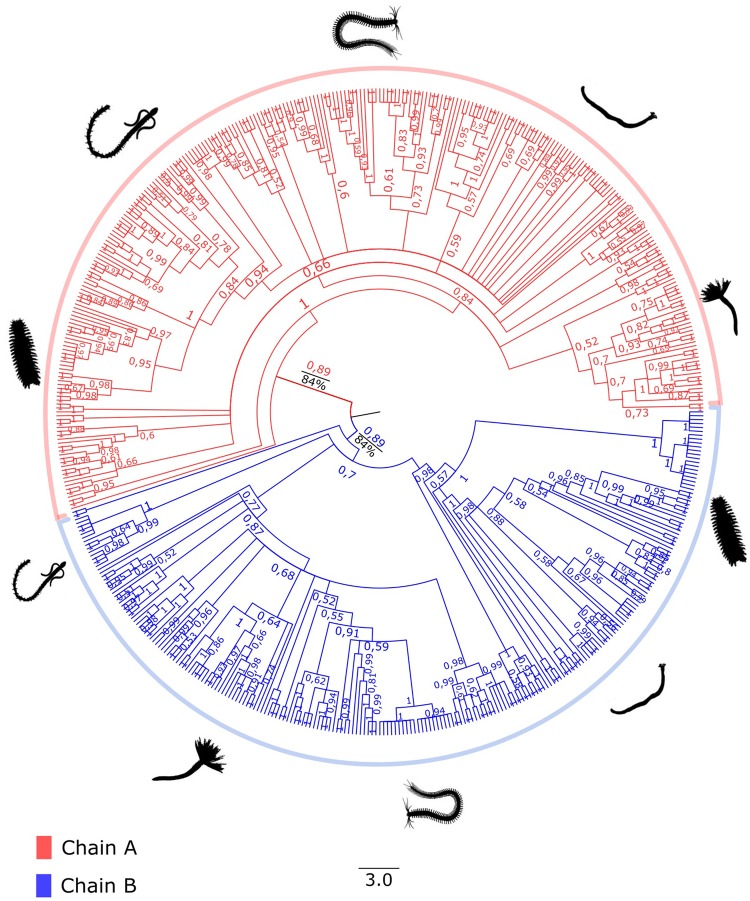
—Bayesian inference gene genealogy of the EG-Ann data set created with MrBayes 3.2.1 (Ronquist and Huelsenbeck 2003) rooted by midpoint of 516 extracellular globin genes from 140 annelid species. The red clade represents the major globin chain A, and the blue clade represents the major globin chain B. Bootstrap support values obtained from the maximum likelihood inference are shown in black, and the posterior probabilities values obtained from the Bayesian inference are shown in red and blue.

**Fig. 3.**
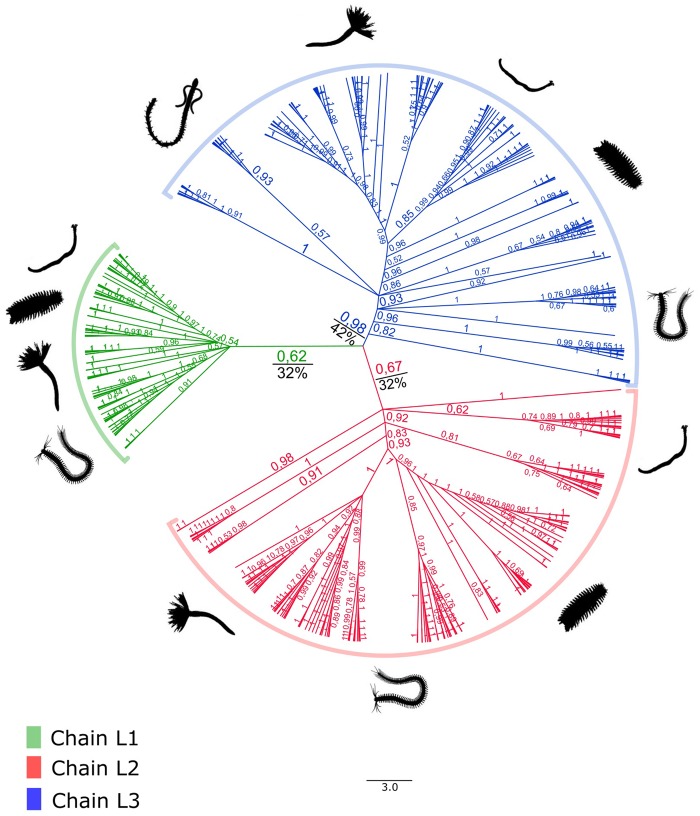
—Bayesian inference gene genealogy of the LIN-Ann data set created with MrBayes 3.2.1 (Ronquist and Huelsenbeck 2003) rooted by midpoint of 387 linker genes from 153 annelid species. The green clade represents the linker chain L1, the red clade represents the linker chain L2, and the blue clade represents the linker chain L3. Bootstrap support values obtained from the maximum likelihood inference are shown in black, and the posterior probabilities values obtained from the Bayesian inference are shown in green, red, and blue.

**Fig. 4.**
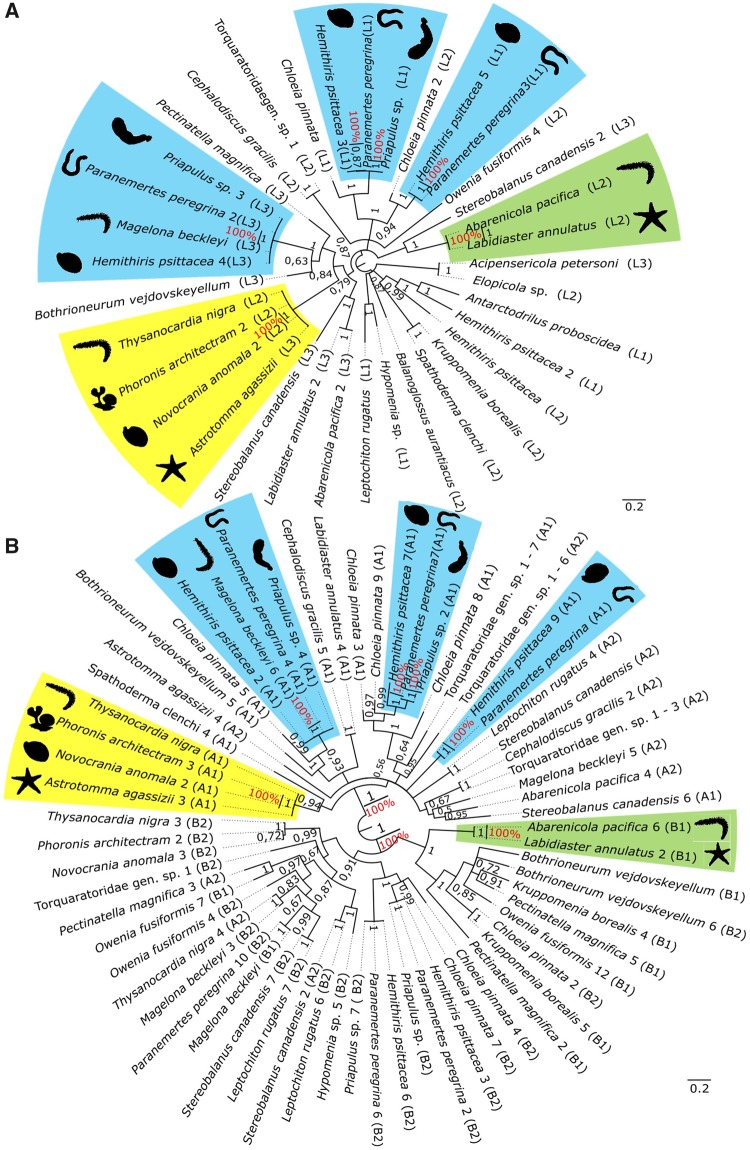
—(*A*) Bayesian inference gene genealogy of the LIN-Met data set created with MrBayes 3.2.1 (Ronquist and Huelsenbeck 2003) rooted by midpoint of 37 linker genes from 26 metazoans. Highlighted boxes correspond to boxes with the same colors in (*B*) indicating same clades that clustered in both metazoan trees. Bootstrap support values obtained from the maximum likelihood inference are shown in red, and the posterior probabilities values obtained from the Bayesian inference are shown in black. (*B*) Bayesian inference gene genealogy of the EG-Met data set created with MrBayes 3.2.1 (Ronquist and Huelsenbeck 2003) rooted by midpoint of 67 extracellular globin genes from 22 metazoans. Highlighted boxes correspond to boxes with the same colors in (*A*) indicating same clades that clustered in both metazoan trees. Bootstrap support values obtained from the maximum likelihood inference are shown in red, and the posterior probabilities values obtained from the Bayesian inference are shown in black.

Two protein data sets were created: the globin data set, comprised all 559 extracellular globin genes retained after the validation steps, and the linker data set, comprised all 414 linker genes retained after the validation steps. Both data sets were independently aligned with MAFFT using the accurate algorithm E-INS-i (Katoh and Standley 2013), and gap-rich regions in the alignments were removed with trimAl 1.2 (Capella-Gutiérrez et al. 2009) using a gap threshold of 0.5 for linker genes and 0.75 for extracellular globin genes (supplementary files 3 and 4, Supplementary Material online). Using Geneious 9.1.3 (Kearse et al. 2012), alignments were visually checked and trimmed to exclude residues 5′ of the putative start codon. Resulting amino acid alignments were used for phylogenetic analyses. Given our data had a predominance of annelid samples, we reconstructed two gene genealogies for both extracellular globin genes and linker genes. For each gene, one tree included all samples from only Annelida and the other spanned all Metazoa samples. The four data sets used to build the trees were as follows: 1) EG-Ann: all 516 extracellular globin sequences which we found in 140 annelid species (fig. 2); 2) LIN-Ann: all 387 linker sequences which we found in 153 annelid species (fig. 3); 3) EG-Met: all 67 extracellular globin sequences of the 22 metazoans in which we found genes distributed in three hemichordates, two echinoderms, two brachiopods, four mollusks, one nemertean, one platyhelminth, one bryozoan, one phoronid, one priapulid, and six annelid species (fig. 4B); and 4) LIN-Met: all 37 linker sequences from the 26 metazoans in which we found genes distributed in two echinoderms, four hemichordates, two brachiopods, three platyhelminths, four mollusks, one nemertean, one bryozoan, one phoronid, one priapulid, and seven annelid species (fig. 4A). The annelids used in both metazoan trees were chosen to represent major families indicative of the breadth of Annelida. ProtTest 3.4 was used to find the best-fit model of protein evolution for the data sets using the Akaike and Bayesian Information Criteria (Darriba et al. 2011). Bayesian phylogenetic inferences were implemented in MrBayes 3.2.1 (Ronquist and Huelsenbeck 2003) with two independent runs, each containing four Metropolis-coupled chains that were run for 10^7^ generations and sampled every 500th generation to approximate posterior distributions. In order to confirm whether chains achieved stationary and determine an appropriate burn-in, we evaluated trace plots of all MrBayes parameter outputs in Tracer v1.6 (Rambaut et al. 2014). The first 25% of samples were discarded as burn-in and a majority rule consensus tree generated using MrBayes. Bayesian posterior probabilities were used for assessing statistical support of each bipartition. The maximum likelihood trees were constructed with RAxML (Kozlov et al. 2018) using the following parameters: The tree topology search was done using the best of BioNJs and NNIs; WAG model for amino acids substitution; uniform substitution rates among sites and bootstrap supports were provided by a 100-replicates. The resultant trees were summarized with FigTree 1.4.3 (Rambaut 2009) and rooted using midpoint rooting (Farris 1972; Hess and Russo 2007). Tertiary structures of extracellular globins and linkers were inferred using the automated protein structure homology-modeling server SWISS-MODEL (Arnold et al. 2006; Kiefer et al. 2009). Representatives of linker and extracellular globin genes from each newly reported phylum were used to confirm the existence of high similarity in tertiary structure (supplementary files 5 and 6, Supplementary Material online).

Additionally, in order to increase phylogenetic coverage, genomes from GenBank were surveyed to verify the presence of linkers and extracellular globins. We employed TBlastN with an *e*-value cutoff of 10^−5^. TBlastN search translated nucleotide databases from NCBI using a protein query (Altschul et al. 1990). We compared the genomes from NCBI of 13 species from eight phyla (Porifera, Cnidaria, Ctenophora, Chordata, Placozoa, Nematoda, Tardigrada, and Arthropoda—supplementary file 7, Supplementary Material online) with two different queries, one comprised five linker sequences from five metazoan species previously selected for possessing HBL-Hbs (MH995534, MH995580, MH995661, MH995788, and MH995796) and another one comprised five extracellular globin sequences from five metazoan species previously selected for possessing HBL-Hbs (MH995926, MH995932, MH996036, MH996080, and MH996186).

## Results

In the initial screening of Trinotate output, we recovered 4,699 nucleotide sequences annotated as extracellular globins and 1,632 nucleotide sequences annotated as linkers. After translation from nucleotides to amino acids and undergoing thorough validation steps such as Pfam domain evaluation, and selection by minimum size, 559 extracellular globin genes and 414 linker genes remained (supplementary file 2, Supplementary Material online). These genes are actively transcribed in 171 species representing ten animal phyla (table 1). Linkers and extracellular globins have not been previously reported from Echinodermata, Hemichordata, Brachiopoda, Mollusca, Nemertea, Bryozoa, Phoronida, Platyhelminthes, and Priapulida. The number of expressed extracellular globin genes in a given species ranged from one in 26 different species to 12 in *Amynthas* sp. (Megascolecidae, Annelida) and *Terebellides stroemii* (Trichobranchidae, Annelida). The number of expressed linker genes ranged from one in 55 different species to 11 in *Randiella* sp. (Randiellidae, Annelida). Transcriptomes from one hemichordate (*Balanoglossus aurantiaca*), two platyhelminths (*Acipensericola petersoni* and *Elopicola* sp.), and 14 annelid species contained only linker genes and lacked extracellular globin genes. Although other species from these phyla presented both globin and linker genes (table 1), as we used transcriptomic data, we can only make inferences about the presence of gene signatures and will not draw conclusions about absences. None of the 13 genomes from GenBank that were surveyed contained either linkers or extracellular globin genes.

**Table 1:** List of All Taxa Analyzed in Which We Found Linkers or/and Extracellular Globins, Including Total Number of Contigs after Assembly, and Number of Linkers and Extracellular Globin Genes

Taxon	Total Number of Contigs	Number of Linker Genes	Accession Number	Number of Globin Genes	Accession Number
Echinodermata					
* Astrotoma agassizii* Lyman, 1875	156,062	1	MH995560	2	MH995909 MH996362
* Labidiaster annulatus* Sladen, 1889	108,871	2	MH995678–79	2	MH996061–62
Hemichordata					
* Balanoglossus aurantiaca* Girard, 1853	143,815	1	MH995569		
* Cephalodiscus gracilis* Harmer, 1905	57,139	1	MH995586	2	MH995925–26
* Stereobalanus canadensis* Spengel, 1893	12,741	2	MH995825–26	4	MH996296–98 MH996416
* *Torquaratoridae gen. sp. 1	102,971	1	MH995851	4	MH996348–51
Annelida					
* Abarenicola pacifica* Healy & Wells, 1959	94,376	2	MH995534 MK011176	2	MH995867–68
* Aeolosoma* sp.	190,647	4	MH995536–39	3	MH995869–71
* Aglaophamus verrilli* (McIntosh, 1885)	118,343	2	MK011177 MH995540	6	MH995872–77
* Alitta succinea* (Leuckart, 1847)	153,011	3	MH995729–30 MK011213	5	MH996143–47
* Amynthas* sp.	18,243	8	MK011182–83 MH995554–59	12	MH995897–908
* Andiorrhinus* sp.	139,858	3	MH995541–43	3	MH995878–80
* Antarctodrilus proboscidea* (Brinkhurst & Fulton, 1979)	49,656	1	MH995544		
* Aphelochaeta* sp.	165,566	5	MK011178–79 MH995545–47	5	MH996356–58 MH995881–82
* Aphrodita japonica* Marenzeller, 1879	120,025	6	MH995548–52 MK011180	7	MH995883–87 MH996359–60
* Arabella* sp.	217,183	1	MH995553	3	MH995888–90
* Aricidea quadrilobata* Webster & Benedict, 1887	81,139	1	MH995864	4	MH995891–93 MH996361
* Armandia* sp.	137,440	1	MK011181	3	MH995894–96
* Auchenoplax crinita* Ehlers, 1887	144,974	5	MH995561–64 MK011184	4	MH995910–13
* Aulodrilus japonicus* Yamaguchi, 1953	109,361	4	MH995565–66 MK011185–86	3	MH995914–15 MH996363
* Axiothella rubrocincta* (Johnson, 1901)	107,215	2	MH995567–68	6	MH995916–20 MH996364
* Bathydrilus rohdei* (Jamieson, 1977)	226,538	4	MH995570–73	1	MH996365
* Bispira pacifica* (Berkeley & Berkeley, 1954)	98,575	2	MH995805–06	7	MH996261–67
* Boccardia proboscidea* Hartman, 1940	117,570	2	MH995574 MK011187		
* Bothrioneurum vejdovskyanum* Štolc, 1886	222,444	1	MH995575	3	MH995921–23
* Branchiobdella kobayashii* Yamaguchi, 1934	56,520	2	MH995576–77		
* Branchiobdella parasita* (Braun, 1805)	39,358	2	MH995578–79		
* Cambarincola gracilis* Robinson, 1954	56,626	2	MH995580–81		
* Cambarincola holti* Hoffman, 1963	46,015	3	MH995582–84		
* Capilloventer* sp.	221,627	2	MH995585 MK011188	2	MH996366 MH995924
* Chaetogaster diaphanus* (Gruithuisen, 1828)	128,034	2	MH995587–88		
* Chaetozone* sp.	143,597	4	MH995589–91 MK011189	5	MH995927–30 MH996367
* Chloeia pinnata* Moore, 1911	130,037	2	MK011190–91	7	MH995931–36 MH996368
* Cirratulus spectabilis* (Kinberg, 1866*)*	120,244	2	MK011192 MH995592	3	MH995937–39
* Cirrodrilus suzukii* (Yamaguchi, 1934)	47,037	3	MH995593–94 MK011193		
* Clymenella torquata* (Leidy, 1855)	111,567	2	MH995595–96	8	MH995940–45 MH996369–70
* Cossura longocirrata* Webster & Benedict, 1887	75,079	5	MH995597–600 MK011194	11	MH995946–56
* Crucigera zygophora* (Johnson, 1901)	116,092	1	MH995601	1	MH995957
* Delaya leruthi* (Hrabĕ, 1958)	118,020	3	MH995602–04	4	MH995958–61
* Dichogaster* green tree worm	116,065	4	MH995605–08	3	MH995962–64
* Dichogaster guadeloupensis* James, 1996	106,438	1	MH995609	3	MH995965–67
* Dichogaster saliens* (Beddard, 1893)	98,665	1	MH995610	1	MH995968
* Diplocardia* sp.	10,235	4	MH995611–13 MK011195	6	MH995969–73 MH996371
* Dodecaceria pulchra* Day, 1955	229,501	3	MH995614–16	5	MH995974–78
* Dorydrilus michaelseni* Piguet, 1913	136,096	2	MH995617–18	3	MH995979–81
* Drawida* sp.	159,219	2	MH995619–20	2	MH995982–83
* Drilocrius* sp.	108,131	4	MH995621–23 MK011196	1	MH995984
* *Echiura gen. sp. green	198,697	1	MH995624	2	MH995985–86
* Eisenia* sp.	168,836	4	MH995856–59	5	MH995987–91
* Enchytraeus albidus* Henle, 1837	22,776	2	MH995626–27	1	MH995992
* Erpobdella octoculata* (Linnaeus, 1758)	59,249	1	MH995628	3	MH995993–95
* Eunice norvegica* (Linnaeus, 1767)	122,784	2	MH995629–30	3	MH995996–98
* Eunice pennata* (Müller, 1776)	93,814	1	MH995631	3	MH995999–6001
* Flabegraviera mundata* (Gravier, 1906)	235,636	1	MH995632	1	MH996002
* Galathowenia oculata* (Zachs, 1923)	179,612	4	MH995633–36	4	MH996003–06
* Galeolaria caespitosa* Lamarck, 1818	143,655	2	MH995637–38	2	MH996007–08
* Gatesona chaetophora* (Bouché, 1972)	104,334	1	MH995639	1	MH996009
* Geogenia benhami* (Rosa, 1891)	84,303	2	MH995640–41	2	MH996010–11
* Glossodrilus* sp.	122,993	2	MK011197 MH995642	1	MH996012
* Glycera dibranchiata* Ehlers, 1868	101,455	1	MH995643	4	MH996013–16
* Glyptonotobdella antarctica* (Sawyer & White, 1969)	64,208	1	MK011198	3	MH996017–19
* Goniada brunnea* Treadwell, 1906	89,398	1	MH995644	2	MH996020–21
* Grania* sp.	68,975	4	MH995860–63	3	MH996022–24
* Guaranidrilus* sp.	105,939	1	MH995645	2	MH996025 MH996372
* Halosydna brevisetosa* Kinberg, 1855	118,418	3	MH995646–48	6	MH996026–30 MH996373
* *Haplotaxidae gen. sp.	100,864	2	MH995649–50	2	MH996031–32
* Haplotaxis gordioides* (Hartmann, 1821)	53,878	1	MH995651	1	MH996033
* Haplotaxis* sp.	93,548	2	MH995652–53	1	MH996034
* Hemigastrodrilus monicae* Bouché, 1970	103,338	5	MH995654–57 MK011199	1	MH996035
* Hermodice carunculata* (Pallas, 1766)	110,813	1	MK011201	3	MH996039–41
* Heronidrilus* sp.	325,567	3	MK011202–03 MH995662	5	MH996042–46
* Hesionides* sp.	219,849	1	MH995663	4	MH996047–50
* Heterodrilus* sp. 1	47,679	4	MK011204–05 MK011252 MH995664	3	MH996051–52 MH996376
* Hrabeiella periglandulata* Pizl and Chalupský, 1984	141,578	2	MH995665–66		
* Idanthyrsus* sp.	201,049	2	MH995668–69	1	MH996053
* Kincaidiana* sp.	83,743	4	MH995670–73	4	MH996054–57
* Komarekiona eatoni* Gates, 1974	143,281	3	MH995674–76	1	MH996058
* Lamellibrachia luymesi* van der Land & Nørrevang, 1975	63,475	2	MH995680–81	10	MH996063–70 MH996378–79
* Laonice* sp.	119,795	1	MH995682	1	MH996071
* Leitoscoloplos robustus* (Verrill, 1873)	219,418	3	MH995683–85	4	MH996072–74 MH996380
* Lepidonotus semitectus* (Stimpson, 1856)	130,020	3	MH995686–87	3	MH996075–77
* Limnodriloides* sp.	151,835	4	MH995689–92	7	MH996081–87
* Lumbriculus variegatus* (Müller, 1774)	109,949	4	MH995693–95 MK011253	4	MH996088–90 MH996381
* Lumbrineris crassicephala* Hartman, 1965	196,426	6	MH995696–700 MK011207	6	MH996091–96
* Lumbrineris perkinsi* Carrera-Parra, 2001	144,648	1	MH995701	3	MH996097–99
* Lutodrilus* sp.	57,341	3	MH995702–04	4	MH996100–03
* Macrochaeta* sp.	230,529	2	MK011208 MH995705	1	MH996382
* Magelona berkeleyi* Jones, 1971	50,123	1	MH995706	4	MH996104–07
* Marphysa sanguinea* (Montagu, 1813)	110,924	1	MH995707	4	MH996108–10 MH996383
* Melinna maculata* Webster, 1879	135,712	5	MH995708–10 MK011209–10	5	MH996111–13 MH996384–85
* Mesenchytraeus pedatus* Eisen, 1904	194,638	2	MH995711–12	2	MH996114–15
* Mesenchytraeus solifugus* DARK (Emery, 1898)	125,494	1	MH995713	1	MH996116
* Mesenchytraeus solifugus* LIGHT (Emery, 1898)	102,971	1	MH995714		
* Mesenchytraeus* sp.	132,680	2	MH995715–16		
* *Microchaetidae gen. sp.	85,460	2	MH995717 MK011211	2	MH996117–18
* Microchaetus* sp.	68,148	1	MH995718	1	MH996119
* Microphthalmus similis* Bobretzky, 1870	169,427	1	MH995719	1	MH996120
* Myxicola infundibulum* (Montagu, 1808)	217,996	4	MH995720–23	6	MH996121–26
* Naineris laevigata* (Grube, 1855)	218,272	3	MH995724–26	11	MH996127–37
* Neosabellaria cementarium* (Moore, 1906)	82,479	1	MK011254	3	MH996138–40
* Nephtys incisa* Malmgren, 1865	188,338	3	MH995727–28 MK011212	4	MH996141–42 MH996386–87
* Nicolea macrobranchia* (Schmarda, 1861)	53,572	2	MH995731–32	1	MH996148
* Nicomache venticola* Blake & Hilbig, 1990	124,708	2	MH995733–34	3	MH996149–50 MH996388
* Ninoe nigripes* Verrill, 1873	151,183	2	MH995735 MK011214	5	MH996151–54 MH996389
* Odontosyllis gibba* Claparède, 1863	131,487	1	MK011215	2	MH996390 MH996157
* Oenone fulgida* (Savigny in Lamarck, 1818)	144,726	2	MK011216 MH995737	4	MH996158–60 MH996391
* Olavius* (*Coralliodriloides*) *loisae* Erséus, 1984	127,965	3	MH995738–39 MK011219	2	MH996162–63
* Olavius albidus* (Jamieson, 1977)	190,000	2	MK011217–18	2	MH996392 MH996161
* *Oligochaeta gen. sp. (unidentified Crassiclitellata—Place Kabary 2)	146,018	4	MH995774–77	5	MH996214–18
* Ophelina acuminata* Örsted, 1843	81,846	2	MH995740–41	5	MH996164–67 MH996393
* Osedax mucofloris* Glover, Kallstrom, Smith & Dahlgren, 2005	40,905	3	MH995742–44	2	MH996168 MH996394
* Owenia fusiformis* Delle Chiaje, 1844	106,476	1	MK011220	3	MH996169–70 MH996395
* Palola* sp.	211,279	1	MH995745	5	MH996171–75
* Parachilota* sp.	72,933	2	MH995746–47	3	MH996176–78
* Paralvinella palmiformis* Desbruyères & Laubier, 1986	85,363	3	MK011221–22 MH995748	2	MH996396–97
* Paramphinome jeffreysii* (McIntosh, 1868)	165,337	5	MK011223–25 MH995749–50	5	MH996179–81 MH996398–99
* Paranais* sp.	100,443	3	MH995751–52 MK011226	2	MH996182 MH996400
* Pectinaria gouldii* (Verrill, 1874)	81,138	5	MH995755–58 MK011228	7	MH996187–93
* Perinereis* sp.	129,117	2	MH995763–64	2	MH996203–04
* Phagodrilus* sp.	80,487	5	MH995765–69	2	MH996205–06
* Pherecardia striata* (Kinberg, 1857)	216,722	2	MH995770 MK011229	3	MH996207–09
* Pherusa plumosa* (Müller, 1776)	170,126	1	MK011230	1	MH996403
* *Phreodrilidae gen. sp. 1	83,059	2	MH995772–73	2	MH996212–13
* Poeobius meseres* Heath, 1930	70,078	3	MH995760–62	6	MH996197–202
* Pontodrilus litoralis* (Grube, 1855)	90,268	6	MH995780–83 MK011231–32	6	MH996224–28 MH996404
* Praxillella pacifica* Berkley, 1929	150,768	3	MK011233–34 MH995784	5	MH996229–33
* Prionospio dubia* Day, 1961	119,949	3	MH995785–86 MK011235	6	MH996234–39
* Propappus volki* Michaelsen, 1916	131,574	2	MH995789–90	3	MH996243–45
* Proscoloplos cygnochaetus* Day, 1954	231,508	2	MH995791 MK011236	4	MH996246–48 MH996406
* Protodriloides chaetifer* (Remane, 1926)	102,702	2	MH995865–66		
* Pseudonereis variegata* (Grube, 1857)	138,332	1	MH995792	1	MH996407
* Randiella* sp.	151,934	11	MH995793–99 MK011237–39 MK011255	4	MH996249–52
* Rhinodrilus priollii* Righi, 1967	87,158	2	MH995800–01	3	MH996253–55
* Rhyacodrilus falciformis* Bretscher, 1901	140,129	4	MH995802–03 MK011256 MK011240	3	MH996256–58
* Sabaco elongatus* (Verrill, 1873)	84,082	1	MH995804	2	MH996259–60
* Scalibregma inflatum* Rathke, 1843	126,107	5	MH995807–10 MK011241	8	MH996268–74 MH996408
* Sclerolinum brattstromi* Webb, 1964	149,694	2	MH995811–12	8	MH996275–80 MH996409–10
* Scolelepis squamata* (Müller, 1806)	147,343	4	MH995813–16	1	MH996411
* Serpula vermicularis* Linnaeus, 1767	151,097	2	MH995818–19	5	MH996283–87
* Siboglinum ekmani* Jägersten, 1956	270,658	1	MH995820	7	MH996288–93 MH996412
* Sparganophilus* sp.	123,905	5	MH995821–24 MK011242	4	MH996413–15 MH996294
* Spirobranchus kraussii* (Baird, 1865)	167,761	2	MH995778–79	5	MH996219–23
* Sternaspis scutata* (Ranzani, 1817)	115,096	3	MK011245–47	9	MH996419–21 MH996303–08
* Sternaspis* sp.	120,636	2	MK011244 MH995827	6	MH996299–302 MH996417–18
* Stygocapitella subterranea* 2 Knöllner, 1934	74,556	2	MH995828–29		
* Stylodrilus heringianus* Claparède, 1862	239,935	4	MH995830–33	4	MH996309–12
* Syllis* cf*. hyalina* Grube, 1863	106,283	4	MH995834–37	9	MH996313–21
* Terebellides stroemii* Sars, 1835	169,760	4	MH995838–40 MK011248	12	MH996322–32 MH996422
* Thalassodrilides* sp.	105,393	1	MH995841	5	MH996333–36 MH996423
* Tharyx kirkegaardi* Blake, 1991	114,157	4	MH995842–44 MK011249	3	MH996337–38 MH996424
* Thelepus crispus* Johnson, 1901	67,478	1	MH995845	1	MH996339
* Thysanocardia nigra* (Ikeda, 1904)	58,011	1	MH995846	3	MH996340–42
* Timarete punctata* (Grube, 1859)	80,306	2	MH995847–48	2	MH996343–44
* Tomopteris* sp.	66,655	2	MH995849–50	3	MH996345–47
* Travisia brevis* Moore, 1923	69,827	1	MK011250	3	MH996352–53 MH996425
* Troglodrilus jugeti* Achurra, Châtelliers & Rodriguez, 2012	157,399	2	MH995852–53	1	MH996354
* Vignysa popi* Bouché, 1970	93,260	2	MH995854–55	1	MH996355
* Xironogiton victoriensis* Gelder and Hall, 1990	55,289	1	MK011251		
Brachiopoda					
* Hemithiris psittacea* (Gmelin, 1790)	103,581	5	MH995658–61 MK011200	5	MH996374–75 MH996036–38
* Novocrania anomala* (O. F. Müller, 1776)	117,369	1	MH995736	2	MH996155–56
Phoronida					
* Phoronis psammophila* Cori, 1889	193,702	1	MH995771	2	MH996210–11
Mollusca					
* Hypomenia* sp.	93,699	1	MH995667	1	MH996377
* Kruppomenia borealis* Odhner, 1920	142,815	1	MH995677	2	MH996059–60
* Leptochiton rugatus* (Carpenter in Pilsbry, 1892)	115,512	1	MH995688	3	MH996078–80
* Spathoderma clenchi* Scheltema, 1985	111,974	1	MK011243	1	MH996295
Nemertea					
* Paranemertes peregrina* Coe, 1901	99,203	3	MH995753–54 MK011227	6	MH996183–86 MH996401–02
Bryozoa					
* Pectinatella magnifica* (Leidy, 1851)	191,465	1	MH995759	3	MH996194–96
Platyhelminthes					
* Acipensericola petersoni* Bullard, Snyder, Jensen & Overstreet, 2008	152,140	1	MH995535		
* Elopicola* sp.	64,384	1	MH995625		
* Selachohemecus olsoni*Short, 1954	135,169	1	MH995817	2	MH996281–82
Priapulida					
* Priapulus* sp.	50,034	2	MH995787–88	4	MH996240–42 MH996405

Note.—GenBank accession numbers are also provided in this table and detailed in supplementary files 8 and 9, Supplementary Material online.

The tertiary structures of linker and extracellular globin genes inferred using the SWISS-MODEL server resulted in proteins with putative respiratory function. Therefore, they could be considered as potentially functional proteins capable of assembling into the HBL-Hb and the presence of complete linkers and extracellular globins was confirmed in each newly recorded phylum. However, using only bioinformatic data, verifying that linkers and globins are assembled and acting as an oxygen-carrying protein complex is not an easy task, but we have taken steps to confirm that the genes under examination possess the required features of functional genes. Complete amino acid alignments of extracellular globin genes had the maximum sequence length of 158 residues, and the alignment of linkers had the maximum length of 236 residues. All extracellular globin sequences started with a methionine and for nearly all the linker sequences the start methionine was recovered, except for *B. aurantiaca* (Hemichordata), *Hypomenia* sp. (Mollusca), *Leptochiton rugatus* (Mollusca), *Spathoderma clenchi* (Mollusca), and *Stereobalanus canadensis* (Hemichordata). Nevertheless, these sequences were maintained due to their high similarity with the remaining linker sequences. All extracellular globin and linker sequences contained their characteristic signature residues, which is a key indicator of potential respiratory function (Gotoh et al. 1987; Yuasa et al. 1996; Negrisolo et al. 2001; Chabasse, Bailly, Rousselot, et al. 2006; Chabasse, Bailly, Sanchez, et al. 2006).

The best-fixed rate model for all data sets was the WAG model. As expected, the EG-Ann gene tree (fig. 2) topology did not mirror the recent Annelida phylogeny (Weigert and Bleidorn 2016). The four paralogous globin types—A1, A2, B1, and B2—were not recovered as monophyletic groups. Only the two main chains originally proposed, A and B, were recovered as clades with strong statistical support (PP > 0.85; fig. 2). In the LIN-Ann gene tree (fig. 3), topological relationships also mismatched annelid phylogeny, supporting the monophyly of each linker chain, L1, L2, and L3 (fig. 3). Both trees representing sampling across metazoans did not reflect the recent phylogenies of Metazoa (Whelan et al. 2015; Halanych 2016; Kocot et al. 2017). In the EG-Met tree, globin chains A and B clustered into two major clades with high support values (PP = 1.0; fig. 4B), but further subdivision into A1/A2 and B1/B2 groups could not be recovered (fig. 4B). In the LIN-Met tree, the three main linker chains did not form monophyletic groups when metazoan sequences were included (fig. 4*A*). Finally, as shown in the metazoan trees (EG-Met and LIN-Met; fig. 4*A* and *B*), highlighted boxes with the same colors (red, blue, yellow, and green clades; fig. 4*A* and *B*) indicate strongly supported clades (PP = 1.0) including the same species in both trees. Although the species in each clade are not closely phylogenetically related, they clustered as sister groups in both metazoan trees with strong statistical support.

## Discussion

**Fig. 5.**
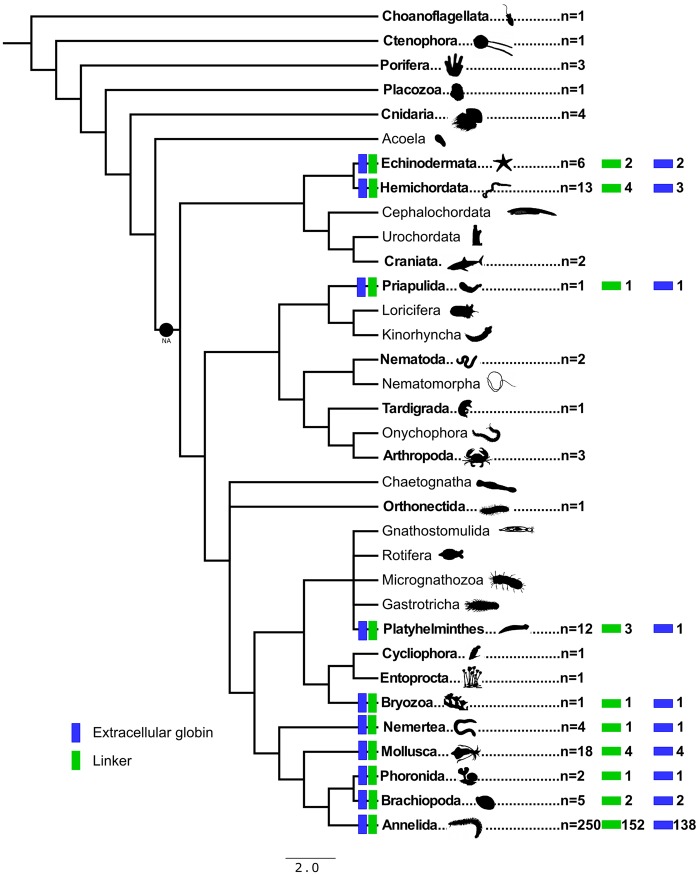
—Hypothesized relationships among metazoan phyla derived from recent phylogenomic studies (Whelan, et al. 2015; Halanych 2016; Kocot, et al. 2017). Names in bold represent analyzed taxa, *n* is the total number of species analyzed in each phylum, blue rectangles represent the number of species with at least one extracellular globin sequence, and green rectangles represent the number of species with at least one linker sequence. NA is nephrozoan ancestor.

Extracellular globin genes and associated linker genes are much more diverse and broadly distributed than previously recognized (Weber and Vinogradov 2001). Herein, actively transcribed linker genes and extracellular globin genes were found in 16 species from nine phyla (other than Annelida) including the first record of extracellular globin genes in deuterostomes. As their distribution was thought to be exclusive to Annelida, all works on evolutionary hypotheses of the emergence of HBL-Hbs suggested that the molecule was already present in, but limited to, the common annelid ancestor (Yuasa et al. 1996; Negrisolo et al. 2001; Bailly 2002). Considering that HBL-Hb oligomers are formed by 144 extracellular globins and 36 linker chains, the independent evolution of this protein complex is unlikely. As no linkers or extracellular globin genes were found in any of the non-nephrozoan genomes surveyed, such as sponges and cnidarians, we suggest that HBL-Hbs must have arisen in the nephrozoan ancestor (fig. 5), just prior to the deuterostome–protostome split. Some phylogenetically uncorrelated species, for example, annelids and starfishes (green boxes, fig. 4*A* and *B*), clustered as sister groups in both EG-Met and LIN-Met trees with high support values, and this seems to be an indication that linkers and extracellular globins may present coevolutionary dynamics.

Goodman et al. (1988) and Hardison (1998) proposed the existence of a common ancestral globin gene that was present before the invertebrate–vertebrate divergence, which is paralogous to the ancestral myoglobin gene. They suggested that if the primitive Hb of metazoans was probably monomeric, thus the multisubunit Hbs represent an independently derived state in Annelida (Goodman et al. 1988). Considering that HBL-Hbs mostly likely appeared in the nephrozoan ancestor, and not in the annelid ancestor, this independently derived state can be extrapolated to nephrozoans. Yuasa et al. (1996) have proposed an evolutionary model for the HBL-Hbs, in which the common ancestor of this molecule was a protein formed only by globin chains, being the linker chains added posteriorly to form the final hexagonal bilayered structure. The work of Shishikura et al. (1986) indicates that the assembly of the extracellular globin trimers which form the globin dodecamer of HBL-Hbs is only possible with the presence of the Cys-19 residue. The free cysteine residue is responsible for the disulfide bond of extracellular globin trimers, which represents the main feature that differs extracellular globins from the intracellular ones (Shishikura et al. 1986; Gotoh et al. 1987). More recently, an additional six “globins” have been identified in vertebrates, namely androglobin, cytoglobin, globin E, globin X, globin Y, and neuroglobin, which form the globin superfamily alongside Hb and myoglobin (reviewed by Burmester and Hankeln 2014). These proteins are structurally conserved but differ in their amino acid sequence compositions and cellular functions, for example, signal transduction, lipid, and nitric oxide metabolism. The ancestor to this protein superfamily is estimated to have arisen some 1.5 Ga (Burmester and Hankeln 2014). These data, alongside our novel observations of giant extracellular Hbs, illustrate a much broader representation and functional repertoire for “globins” in extant species.

According to the pioneering work of Gotoh et al. (1987), the gene duplication events that gave rise to the two paralogous extracellular globin chains A and B occurred before the Annelida diversification. They suggest that the two paralogous extracellular globin genes arose from a single ancient duplication event, comparable to the α and β chains from the vertebrate Hbs (Gotoh et al. 1987). Although later works recovered clades A and B (Negrisolo et al. 2001; Bailly 2002; Chabasse, Bailly, Rousselot, et al. 2006), they failed to support further subdivisions A1, A2, B1, and B2 as monophyletic groups, which agrees with our findings. Thus, our results confirmed the early hypothesis of two main paralogous globin chains, A and B (Gotoh et al. 1987), both in annelids and other metazoans. Further subdivisions (A1/A2 and B1/B2) though were not consistently recovered, either in annelids or in other metazoans. Regarding linkers, the works of Suzuki et al. (1990) and Suzuki and Riggs (1993) described three linker chains among annelids (L1, L2, and L3). Negrisolo et al. (2001), analyzing seven linker sequences, were unable to recover the three linker subtypes as monophyletic groups. Using 22 linker sequences, Chabasse, Bailly, Sanchez, et al. (2006) clustered linkers in two main clades but with low statistical support values. Employing more than 400 linker sequences, our much larger sample generated results that support the existence of three linker types L1, L2, and L3 among Annelida in agreement with Suzuki et al. (1990) and Suzuki and Riggs (1993). However, when other metazoan taxa were included in the analyses, none of the three linker types could be recovered as monophyletic groups. We corroborate the findings of three linker groups for annelids; however, this does not appear to be the case for higher taxonomic levels. Although the three linker clades L1, L2, and L3 presented low bootstrap values in the LIN-Ann gene tree (fig. 3), we considered these groups to be valid because they have already been widely recorded and discussed in the literature in annelid species (Suzuki et al. 1990; Suzuki and Riggs 1993; Royer et al. 2006), and the posterior probability values have also corroborated the monophyly of the three groups (fig. 3). Moreover, our findings confirmed that Annelida harbors the greatest diversity of oxygen-carrying proteins among all other animal phyla (Costa-Paiva et al. 2017, 2018). Like Hrs and Hcs, the expansion of the extracellular globins in invertebrates may be associated with diverse biological functions other than oxygen transport. For example, Hrs and Hcs participate in metal detoxification (cadmium) and innate immunity, respectively. Hbs, including those from humans, blood clams (e.g., *Telligarca granosa*), alligators, and fish are precursors of antimicrobial peptides and act as enzymatic antioxidants under certain conditions in vivo and in vitro (Coates and Decker 2017).

Lamy et al. (2000) suggested that the presence of the three linker types is not required for the assembly of the HBL-Hb molecule. Nevertheless, they showed that every linker chain can replace each other in the HBL-Hb assembly, and the molecule can assemble with only one or two types of linker chains. Their results also suggest that the globin dodecamer is unstable without linkers (Lamy et al. 2000). Additionally, the inferred tertiary structures of the novel extracellular globins suggested that they could have a putative respiratory function (supplementary file 5, Supplementary Material online). Using only bioinformatic data, we cannot state that the large complex structure of HBL-Hbs is the same in all metazoan lineages, which could be a unique adaptation within Annelida. Considering that the role of the linker subunits is predominantly structural, because in their absence, the functional globin dodecamer does not assemble into the hexagonal bilayer structure (Lamy et al. 1996), we believe this is sufficient evidence that the structure of large HBL-Hb complexes made of linkers and globins is a feature also present in other metazoans besides annelids. Because linkers can replace each other and the subdivision of extracellular globins into A1/A2 and B1/B2 groups appears to be largely descriptive, we argue that species expressing extracellular globins and linkers could be capable of assembling the subunits into HBL-Hbs to facilitate oxygen transportation.

Our findings demonstrate that the diversity of HBL-Hbs is much greater than traditionally assumed and is found in multiple metazoan lineages, and both linkers and extracellular globins were likely present in the nephrozoan ancestor. The comprehensive phylogenetic analyses of transcriptomic data from >100 metazoans corroborated the results of Gotoh et al. (1987), which classified extracellular globins into two major chains (A and B). Conversely, our data did not support the subdivision of extracellular globins into A1/A2 and B1/B2 groups, indicating that they are not natural subfamilies. Also, our data supported the subdivision of linker units into L1, L2, and L3 in annelids only. Although the reconstructed tertiary structures of novel extracellular globin have shown the presence of putative oxygen-binding sites, additional studies on the biochemical properties of HBL-Hbs within the newly recorded groups would be the next step for confirming their oxygen-carrying capabilities.

## Supplementary Material

Supplementary Data
