# Supplementary material for: Newly Discovered Occurrences and Gene Tree of the Extracellular Globins and Linker Chains from the Giant Hexagonal Bilayer Hemoglobin in Metazoans
Source: Genome Biol Evol. 2019 Jan 21;11(3):597–612. doi: 10.1093/gbe/evz012 (PMC6400237; doi:10.1093/gbe/evz012)
Supplement: Supplementary Data [file evz012_supp.zip › Supplementary_file1.docx]

**Supplementary file 1 -** List of all taxa analyzed, including total number of contigs after assembly, and number of linkers and extracellular globin genes, and their respective GenBank accession numbers. Gene numbers marked with asterisks represent partial sequences not used in the analyzes.

| **Taxon** | **Total contigs number** | **Linker Genes Number** | **Accession number** | **Globin genes number** | **Accession number** |
| --- | --- | --- | --- | --- | --- |
| **CHOANOFLAGELLATA** |  |  |  |  |  |
| *Salpingoeca pyxidium* Kent, 1881 | 202,399 | 2* |  |  |  |
| **METAZOA** |  |  |  |  |  |
| **Porifera** |  |  |  |  |  |
| *Kirkpatrickia variolosa* (Kirkpatrick, 1907) | 100,231 | 1* |  |  |  |
| *Latrunculia apicalis* Ridley and Dendy, 1886 | 76,210 |  |  |  |  |
| **Cnidaria** |  |  |  |  |  |
| *Gersemia antarctica* (Kukenthal, 1902) | 20,023 |  |  |  |  |
| Staurozoa gen. sp. | 45,023 |  |  |  |  |
| **Echinodermata** |  |  |  |  |  |
| *Astrotoma agassizii* Lyman, 1875 | 156,062 | 1 | MH995560 | 2 | MH995909 MH996362 |
| *Labidiaster annulatus* Sladen, 1889 | 108,871 | 2 | MH995678-79 | 2 | MH996061-62 |
| *Labidiaster* sp. | 168,72 |  |  |  |  |
| *Leptosynapta clarki* Heding, 1928 | 242,126 |  |  |  |  |
| *Apostichopus californicus* (Stimpson, 1857) | 134,64 |  |  |  |  |
| Crinoidea gen. sp. | 127,039 |  |  |  |  |
| **Hemichordata** |  |  |  |  |  |
| *Balanoglossus aurantiaca* Girard, 1853 | 143,815 | 1 | MH995569 |  |  |
| *Cephalodiscus gracilis* Harmer, 1905 | 57,139 | 1 | MH995586 | 2 | MH995925-26 |
| *Cephalodiscus hodgsoni* Ridewood, 1907 | 200,052 |  |  |  |  |
| *Cephalodiscus nigrescens* Lankester, 1905 | 11,565 |  |  |  |  |
| Harrimaniidae gen. sp. (from Iceland) | 230,054 |  |  |  |  |
| Harrimaniidae gen. sp. (from Norway) | 274,434 |  |  |  |  |
| *Ptychodera bahamensis* Spengel, 1893 | 115,310 |  |  |  |  |
| *Rhabdopleura* sp. | 4,790 |  |  |  |  |
| *Saccoglossus mereschkowskii* Wagner, 1885 | 145,937 |  |  |  |  |
| *Schizocardium brasiliense* Spengel, 1893 | 101,493 |  |  |  |  |
| *Stereobalanus canadensis* Spengel, 1893 | 12,741 | 2 | MH995825-26 | 4 | MH996296-98 MH996416 |
| Torquaratoridae gen. sp. 1 | 102,971 | 1 | MH995851 | 4 | MH996348-51 |
| Torquaratoridae gen. sp. 2 | 145,544 |  |  |  |  |
| **Brachiopoda** |  |  |  |  |  |
| *Glottidia pyramidata* (Stimpson, 1860) | 131,562 |  |  |  |  |
| *Hemithiris psittacea* (Gmelin, 1790) | 103,581 | 5 | MH995658-61 MK011200 | 5 | MH996374-75 MH996036-38 |
| *Laqueus californicus* (Koch, 1848) | 133,086 |  |  |  |  |
| *Macandrevia cranium* (O. F. Müller, 1776) | 9,695 |  |  |  |  |
| *Novocrania anomala* (O. F. Müller, 1776) | 117,369 | 1 | MH995736 | 2 | MH996155-56 |
| **Phoronida** |  |  |  |  |  |
| *Phoronis psammophila* Cori, 1889 | 193,702 | 1 | MH995771 | 2 | MH996210-11 |
| *Phoronopsis harmeri* Pixell, 1912 | 283,821 |  |  |  |  |
| **Mollusca** |  |  |  |  |  |
| Aculifera gen. sp. | 109,736 |  |  |  |  |
| *Alexandromenia crassa* Odhner, 1920 | 111,729 |  |  |  |  |
| Amphimeniidae gen. sp. | 130,196 |  |  |  |  |
| *Cavibelonia* sp. | 144,105 |  |  |  |  |
| *Entonomenia tricarinata* (Salvini-Plawen, 1978) | 147,128 |  |  |  |  |
| *Epimenia babai* Salvini-Plawen, 1997 | 71,819 |  |  |  |  |
| *Helluoherpia aegiri* Handl and Buchinger, 1996 | 95,935 |  |  |  |  |
| *Hypomenia* sp*.* | 93,699 | 1 | MH995667 | 1 | MH996377 |
| *Kruppomenia borealis* Odhner, 1920 | 142,815 | 1 | MH995677 | 2 | MH996059-60 |
| *Leptochiton rugatus* (Carpenter in Pilsbry, 1892) | 115,512 | 1 | MH995688 | 3 | MH996078-80 |
| *Macellomenia* sp. | 107,525 |  |  |  |  |
| *Meiomenia swedmarki* Morse, 1979 | 118,867 |  |  |  |  |
| *Neomenia carinata* Tullberg, 1875 | 172,727 |  |  |  |  |
| *Phyllomenia* sp. | 170,739 |  |  |  |  |
| *Prochaetoderma californicum* Schwabl, 1963 | 293,209 |  |  |  |  |
| *Scutopus ventrolineatus* Salvini-Plawen, 1968 | 221,900 |  |  |  |  |
| *Simrothiella margaritacea* (Koren and Danielssen, 1877) | 99,722 |  |  |  |  |
| *Spathoderma clenchi* Scheltema, 1985 | 111,974 | 1 | MK011243 | 1 | MH996295 |
| **Nemertea** |  |  |  |  |  |
| *Malacobdella grossa* (Müller, 1779) | 79,313 |  |  |  |  |
| *Paranemertes peregrina* Coe, 1901 | 99,203 | 3 | MH995753-54 MK011227 | 6 | MH996183-86 MH996401-02 |
| *Parborlasia corrugatus* (McIntosh, 1876) | 911,662 |  |  |  |  |
| *Tubulanus polymorphus* Renier, 1804 | 109,120 |  |  |  |  |
| **Bryozoa** |  |  |  |  |  |
| *Pectinatella magnifica* (Leidy, 1851) | 191,465 | 1 | MH995759 | 3 | MH996194-96 |
| **Cycliophora** |  |  |  |  |  |
| *Symbion americanus* Obst, Funch and Kristensen, 2006 | 135,725 |  |  |  |  |
| **Entoprocta** |  |  |  |  |  |
| *Loxosoma pectinaricola* Franzen, 1962 | 144,339 | 1* |  |  |  |
| **Platyhelminthes** |  |  |  |  |  |
| *Acipensericola petersoni* Bullard, Snyder, Jensen & Overstreet, 2008 | 152,140 | 1 | MH995535 |  |  |
| *Cardicola currani* Bullard and Overstreet, 2004 | 86,962 |  |  |  |  |
| *Cardicola palmeri* Bullard and Overstreet, 2004 | 52,837 |  |  |  |  |
| *Elaphrobates euzeti* Bullard and Overstreet, 2003 | 118,013 |  |  |  |  |
| *Elopicola* sp. | 64,384 | 1 | MH995625 |  |  |
| *Hapalorhynchus* sp. | 42,863 |  |  |  |  |
| *Myliobaticola richardheardi* Bullard and Jensen, 2008 | 15,147 |  |  |  |  |
| *Myliobaticola* sp. | 73,883 |  |  |  |  |
| *Sanguinicola* sp. | 39,616 |  |  |  |  |
| Platyhelminthes gen. sp. (Elops parasite) | 222,375 |  |  |  |  |
| *Psettarium anthicum* Bullard and Overstreet, 2006 | 39,616 |  |  |  |  |
| *Selachohemecus olsoni*Short, 1954 | 135,169 | 1 | MH995817 | 2 | MH996281-82 |
| **Orthonectida** |  |  |  |  |  |
| Orthonectida gen. sp. | 231,032 |  |  |  |  |
| **Priapulida** |  |  |  |  |  |
| *Priapulus* sp*.* | 50,034 | 2 | MH995787-88 | 4 | MH996240-42 MH996405 |
| **Annelida** |  |  |  |  |  |
| *Abarenicola pacifica* Healy & Wells, 1959 | 94,376 | 2 | MH995534 MK011176 | 2 | MH995867-68 |
| Acanthodrilidae gen. sp. | 181,228 |  |  |  |  |
| *Aeolosoma* sp*.* | 190,647 | 4 | MH995536-39 | 3 | MH995869-71 |
| *Aglaophamus verrilli* (McIntosh, 1885) | 118,343 | 2 | MK011177 MH995540 | 6 | MH995872-77 |
| *Alciopa* sp. | 233,051 |  |  |  |  |
| *Alitta succinea* (Leuckart, 1847) | 153,011 | 3 | MH995729-30 MK011213 | 5 | MH996143-47 |
| *Alma* sp. | 110,015 |  |  |  |  |
| *Amphisamytha galapagensis* Zottoli, 1983 | 14,313 |  |  |  |  |
| *Amynthas* sp. | 18,243 | 8 | MK011182-83 MH995554-59 | 12 | MH995897-908 |
| *Ancistrosyllis groenlandica* McIntosh, 1878 | 94,924 |  |  |  |  |
| *Andiorrhinus* sp. | 139,858 | 3 | MH995541-43 | 3 | MH995878-80 |
| *Ankyrodrilus legaeus* Holt, 1965 | 54,246 |  |  |  |  |
| Annelida gen. sp. (unidentified EW1 annelid) | 172,934 |  |  |  |  |
| *Antarctodrilus proboscidea* (Brinkhurst & Fulton, 1979) | 49,656 | 1 | MH995544 |  |  |
| *Aphelochaeta* sp. | 165,566 | 5 | MK011178-79 MH995545-47 | 5 | MH996356-58 MH995881-82 |
| *Aphrodita japonica* Marenzeller, 1879 | 120,025 | 6 | MH995548-52 MK011180 | 7 | MH995883-87 MH996359-60 |
| *Arabella* sp*.* | 217,183 | 1 | MH995553 | 3 | MH995888-90 |
| *Areco reco* Righi, Ayres & Bittencourt, 1978 | 170,510 |  |  |  |  |
| *Arenicola loveni* Kinberg, 1866 | 27,028 |  |  |  |  |
| *Arhynchite pugettensis* Fisher, 1949 | 20,724 |  |  |  |  |
| *Arichlidon gathofi* Watson Russell, 2000 | 140,98 |  |  |  |  |
| *Aricidea quadrilobata* Webster & Benedict, 1887 | 81,139 | 1 | MH995864 | 4 | MH995891-93 MH996361 |
| *Armandia* sp*.* | 137,440 | 1 | MK011181 | 3 | MH995894-96 |
| *Aspidosiphon laevis* Quatrefages, 1865 | 168,072 |  |  |  |  |
| *Auchenoplax crinita* Ehlers, 1887 | 144,974 | 5 | MH995561-64 MK011184 | 4 | MH995910-13 |
| *Aulodrilus japonicus* Yamaguchi, 1953 | 109,361 | 4 | MH995565-66 MK011185-86 | 3 | MH995914-15 MH996363 |
| *Autolytus tuberculatus* (Schmarda, 1861) | 137,934 |  |  |  |  |
| *Avelona ligra* (Bouche, 1969) | 182,509 |  |  |  |  |
| *Axiothella rubrocincta* (Johnson, 1901) | 107,215 | 2 | MH995567-68 | 6 | MH995916-20 MH996364 |
| *Bathydrilus rohdei* (Jamieson, 1977) | 226,538 | 4 | MH995570-73 | 1 | MH996365 |
| *Bdellodrilus illuminatus* (Moore, 1894) | 67,562 |  |  |  |  |
| *Bhawania goodei* Webster, 1884 | 70,615 |  |  |  |  |
| *Bispira pacifica* (Berkeley & Berkeley, 1954) | 98,575 | 2 | MH995805-06 | 7 | MH996261-67 |
| *Boccardia proboscidea* Hartman, 1940 | 117,570 | 2 | MH995574 MK011187 |  |  |
| *Bothrioneurum vejdovskyanum* Štolc, 1886 | 222,444 | 1 | MH995575 | 3 | MH995921-23 |
| *Branchiobdella kobayashii* Yamaguchi, 1934 | 56,520 | 2 | MH995576-77 |  |  |
| *Branchiobdella parasita* (Braun, 1805) | 39,358 | 2 | MH995578-79 |  |  |
| *Cambarincola gracilis* Robinson, 1954 | 56,626 | 2 | MH995580-81 |  |  |
| *Cambarincola holti* Hoffman, 1963 | 46,015 | 3 | MH995582-84 |  |  |
| *Capilloventer* sp. | 221,627 | 2 | MH995585 MK011188 | 2 | MH996366 MH995924 |
| *Chaetacanthus magnificus* (Grube, 1876) | 95,443 |  |  |  |  |
| *Chaetogaster diaphanus* (Gruithuisen, 1828) | 128,034 | 2 | MH995587-88 |  |  |
| *Chaetopterus variopedatus* (Renier, 1804) | 147,132 |  |  |  |  |
| *Chaetozone* sp. | 143,597 | 4 | MH995589-91 MK011189 | 5 | MH995927-30 MH996367 |
| *Chloeia pinnata* Moore, 1911 | 130,037 | 2 | MK011190-91 | 7 | MH995931-36 MH996368 |
| *Chone* sp. | 106,577 |  |  |  |  |
| *Cirratulus spectabilis* (Kinberg, 1866*)* | 120,244 | 2 | MK011192 MH995592 | 3 | MH995937-39 |
| *Cirrodrilus suzukii* (Yamaguchi, 1934) | 47,037 | 3 | MH995593-94 MK011193 |  |  |
| *Clymenella torquata* (Leidy, 1855) | 111,567 | 2 | MH995595-96 | 8 | MH995940-45 MH996369-70 |
| *Cossura longocirrata* Webster & Benedict, 1887 | 75,079 | 5 | MH995597-600 MK011194 | 11 | MH995946-56 |
| *Criodrilus lacuum* Hoffmeister, 1845 | 119,084 |  |  |  |  |
| *Crucigera zygophora* (Johnson, 1901) | 116,092 | 1 | MH995601 | 1 | MH995957 |
| *Ctenodrilus* sp. | 373,077 |  |  |  |  |
| Cylicobdellidae gen. sp. | 53,875 |  |  |  |  |
| *Delaya leruthi* (Hrabĕ, 1958) | 118,020 | 3 | MH995602-04 | 4 | MH995958-61 |
| *Dendrobaena hortensis* (Michaelsen, 1890) | 179,981 |  |  |  |  |
| *Dichogaster* green tree worm | 116,065 | 4 | MH995605-08 | 3 | MH995962-64 |
| *Dichogaster guadeloupensis* James, 1996 | 106,438 | 1 | MH995609 | 3 | MH995965-67 |
| *Dichogaster saliens* (Beddard, 1893) | 98,665 | 1 | MH995610 | 1 | MH995968 |
| *Dinophilus gyrociliatus* O. Schmidt, 1857 | 188,729 |  |  |  |  |
| *Diopatra cuprea* (Bosc, 1802) | 138,779 |  |  |  |  |
| *Diplocardia* sp. | 10,235 | 4 | MH995611-13 MK011195 | 6 | MH995969-73 MH996371 |
| *Dodecaceria pulchra* Day, 1955 | 229,501 | 3 | MH995614-16 | 5 | MH995974-78 |
| *Dorvillea* sp. | 150,550 |  |  |  |  |
| *Dorydrilus michaelseni* Piguet, 1913 | 136,096 | 2 | MH995617-18 | 3 | MH995979-81 |
| *Drawida* sp. | 159,219 | 2 | MH995619-20 | 2 | MH995982-83 |
| *Drilocrius* sp*.* | 108,131 | 4 | MH995621-23 MK011196 | 1 | MH995984 |
| Drilonereis sp | 12,598 |  |  |  |  |
| Echiura gen. sp. geach | 200,454 |  |  |  |  |
| Echiura gen. sp. green | 198,697 | 1 | MH995624 | 2 | MH995985-86 |
| *Eisenia* sp*.* | 168,836 | 4 | MH995856-59 | 5 | MH995987-91 |
| *Enchytraeus albidus* Henle, 1837 | 22,776 | 2 | MH995626-27 | 1 | MH995992 |
| *Erpobdella octoculata* (Linnaeus, 1758) | 59,249 | 1 | MH995628 | 3 | MH995993-95 |
| *Eteone* sp. | 41,912 |  |  |  |  |
| *Eudrilus eugeniae* (Kinberg, 1867) | 85,990 |  |  |  |  |
| *Eulalia myriacyclum* (Schmarda, 1861) | 110,762 |  |  |  |  |
| *Eunice norvegica* (Linnaeus, 1767) | 122,784 | 2 | MH995629-30 | 3 | MH995996-98 |
| *Eunice pennata* (Müller, 1776) | 93,814 | 1 | MH995631 | 3 | MH995999-6001 |
| *Euphrosine capensis* Kinberg, 1857 | 72,22 |  |  |  |  |
| *Eupolymnia nebulosa* (Montagu, 1819) | 139,021 |  |  |  |  |
| *Exallopus* sp. | 55,654 |  |  |  |  |
| *Fauveliopsis scabra* Hartman & Fauchald, 1971 | 161,926 |  |  |  |  |
| *Fimoscolex* sp. | 95,465 |  |  |  |  |
| *Flabegraviera mundata* (Gravier, 1906) | 235,636 | 1 | MH995632 | 1 | MH996002 |
| Flabelligeridae gen. sp. | 157,924 |  |  |  |  |
| *Galathowenia oculata* (Zachs, 1923) | 179,612 | 4 | MH995633-36 | 4 | MH996003-06 |
| *Galeolaria caespitosa* Lamarck, 1818 | 143,655 | 2 | MH995637-38 | 2 | MH996007-08 |
| *Gatesona chaetophora* (Bouché, 1972) | 104,334 | 1 | MH995639 | 1 | MH996009 |
| *Gattyana cirrhosa* (Pallas, 1766) | 188,001 |  |  |  |  |
| *Geogenia benhami* (Rosa, 1891) | 84,303 | 2 | MH995640-41 | 2 | MH996010-11 |
| *Glossodrilus* sp*.* | 122,993 | 2 | MK011197 MH995642 | 1 | MH996012 |
| *Glossoscolex* sp. | 58,411 |  |  |  |  |
| *Glycera americana* Leidy, 1855 | 126,229 |  |  |  |  |
| *Glycera dibranchiata* Ehlers, 1868 | 101,455 | 1 | MH995643 | 4 | MH996013-16 |
| *Glycinde armigera* Moore, 1911 | 79,528 |  |  |  |  |
| *Glyptonotobdella antarctica* (Sawyer & White, 1969) | 64,208 | 1 | MK011198 | 3 | MH996017-19 |
| *Goniada brunnea* Treadwell, 1906 | 89,398 | 1 | MH995644 | 2 | MH996020-21 |
| *Grania* sp*.* | 68,975 | 4 | MH995860-63 | 3 | MH996022-24 |
| *Guaranidrilus* sp*.* | 105,939 | 1 | MH995645 | 2 | MH996025 MH996372 |
| *Haemopis sanguisuga* (Linnaeus, 1758) | 84,154 |  |  |  |  |
| *Halosydna brevisetosa* Kinberg, 1855 | 118,418 | 3 | MH995646-48 | 6 | MH996026-30 MH996373 |
| Haplotaxidae gen. sp. | 100,864 | 2 | MH995649-50 | 2 | MH996031-32 |
| *Haplotaxis gordioides* (Hartmann, 1821) | 53,878 | 1 | MH995651 | 1 | MH996033 |
| *Haplotaxis* sp. | 93,548 | 2 | MH995652-53 | 1 | MH996034 |
| *Harmothoe oculinarum* (Storm, 1879) | 94,991 |  |  |  |  |
| *Hemigastrodrilus monicae* Bouché, 1970 | 103,338 | 5 | MH995654-57 MK011199 | 1 | MH996035 |
| *Hemipodia simplex* (Grube, 1857) | 55,653 |  |  |  |  |
| *Hermenia verruculosa* Grube, 1856 | 111,026 |  |  |  |  |
| *Hermodice carunculata* (Pallas, 1766) | 110,813 | 1 | MK011201 | 3 | MH996039-41 |
| *Heronidrilus* sp. | 325,567 | 3 | MK011202-03 MH995662 | 5 | MH996042-46 |
| *Hesionides* sp*.* | 219,849 | 1 | MH995663 | 4 | MH996047-50 |
| *Heterodrilus* sp. 1 | 47,679 | 4 | MK011204-05 MK011252 MH995664 | 3 | MH996051-52 MH996376 |
| *Heterodrilus* sp. 2 | 74,803 |  |  |  |  |
| *Heteromastus filiformis* (Claparède, 1864) | 148,196 |  |  |  |  |
| *Histriobdella homari* Beneden, 1858 | 143,130 |  |  |  |  |
| *Hrabeiella periglandulata* Pizl and Chalupský, 1984 | 141,578 | 2 | MH995665-66 |  |  |
| *Idanthyrsus* sp. | 201,049 | 2 | MH995668-69 | 1 | MH996053 |
| *Ilyphagus octobranchus* Hartman, 1965 | 189,641 |  |  |  |  |
| *Kazimierzus* sp. | 251,294 |  |  |  |  |
| *Kerriona* sp. | 104,982 |  |  |  |  |
| *Kincaidiana* sp. | 83,743 | 4 | MH995670-73 | 4 | MH996054-57 |
| *Komarekiona eatoni* Gates, 1974 | 143,281 | 3 | MH995674-76 | 1 | MH996058 |
| *Kynotus pittarellii* Cognetti, 1906 | 108,836 |  |  |  |  |
| *Laetmonice producta* Grube, 1877 | 73,530 |  |  |  |  |
| *Lamellibrachia luymesi* van der Land & Nørrevang, 1975 | 63,475 | 2 | MH995680-81 | 10 | MH996063-70 MH996378-79 |
| *Lanicides* sp. | 129,689 |  |  |  |  |
| *Laonice* sp. | 119,795 | 1 | MH995682 | 1 | MH996071 |
| *Leanira* sp. | 115,908 |  |  |  |  |
| *Leitoscoloplos robustus* (Verrill, 1873) | 219,418 | 3 | MH995683-85 | 4 | MH996072-74 MH996380 |
| *Lepidonotus semitectus* (Stimpson, 1856) | 130,020 | 3 | MH995686-87 | 3 | MH996075-77 |
| *Limnodriloides* sp*.* | 151,835 | 4 | MH995689-92 | 7 | MH996081-87 |
| *Lithacrosiphon cristatus* (Sluiter, 1902) | 136,386 |  |  |  |  |
| *Lumbriculus variegatus* (Müller, 1774) | 109,949 | 4 | MH995693-95 MK011253 | 4 | MH996088-90 MH996381 |
| *Lumbrineris crassicephala* Hartman, 1965 | 196,426 | 6 | MH995696-700 MK011207 | 6 | MH996091-96 |
| *Lumbrineris perkinsi* Carrera-Parra, 2001 | 144,648 | 1 | MH995701 | 3 | MH996097-99 |
| *Lutodrilus* sp. | 57,341 | 3 | MH995702-04 | 4 | MH996100-03 |
| *Lysilla* sp. | 104,324 |  |  |  |  |
| *Macrochaeta* sp. | 230,529 | 2 | MK011208 MH995705 | 1 | MH996382 |
| *Magelona berkeleyi* Jones, 1971 | 50,123 | 1 | MH995706 | 4 | MH996104-07 |
| *Maoridrilus wilkini* Lee, 1959 | 80,910 |  |  |  |  |
| *Marphysa sanguinea* (Montagu, 1813) | 110,924 | 1 | MH995707 | 4 | MH996108-10 MH996383 |
| *Melinna maculata* Webster, 1879 | 135,712 | 5 | MH995708-10 MK011209-10 | 5 | MH996111-13 MH996384 -85 |
| *Mesenchytraeus pedatus* Eisen, 1904 | 194,638 | 2 | MH995711-12 | 2 | MH996114-15 |
| *Mesenchytraeus solifugus* DARK (Emery, 1898) | 125,494 | 1 | MH995713 | 1 | MH996116 |
| *Mesenchytraeus solifugus* LIGHT (Emery, 1898) | 102,971 | 1 | MH995714 |  |  |
| *Mesenchytraeus* sp. | 132,680 | 2 | MH995715-16 |  |  |
| *Mesochaetopterus taylori* Potts, 1914 | 83,209 |  |  |  |  |
| Microchaetidae gen. sp. | 85,460 | 2 | MH995717 MK011211 | 2 | MH996117-18 |
| *Microchaetus* sp. | 68,148 | 1 | MH995718 | 1 | MH996119 |
| *Microphthalmus listensis* Westheide, 1967 | 58,792 |  |  |  |  |
| *Microphthalmus similis* Bobretzky, 1870 | 169,427 | 1 | MH995719 | 1 | MH996120 |
| *Myxicola infundibulum* (Montagu, 1808) | 217,996 | 4 | MH995720-23 | 6 | MH996121-26 |
| *Naineris laevigata* (Grube, 1855) | 218,272 | 3 | MH995724-26 | 11 | MH996127-37 |
| *Neosabellaria cementarium* (Moore, 1906) | 82,479 | 1 | MK011254 | 3 | MH996138-40 |
| *Nephasoma flagriferum* (Selenka, 1885) | 170,216 |  |  |  |  |
| *Nephtys incisa* Malmgren, 1865 | 188,338 | 3 | MH995727-28 MK011212 | 4 | MH996141-42 MH996386-87 |
| *Nereiphylla* sp. | 131,047 |  |  |  |  |
| *Nerilla antennata* Schmidt, 1848 | 65,078 |  |  |  |  |
| *Nicolea macrobranchia* (Schmarda, 1861) | 53,572 | 2 | MH995731-32 | 1 | MH996148 |
| *Nicomache venticola* Blake & Hilbig, 1990 | 124,708 | 2 | MH995733-34 | 3 | MH996149-50 MH996388 |
| *Ninoe nigripes* Verrill, 1873 | 151,183 | 2 | MH995735 MK011214 | 5 | MH996151-54 MH996389 |
| *Notomastus tenuis* Moore, 1909 | 129,745 |  |  |  |  |
| *Odontosyllis gibba* Claparède, 1863 | 131,487 | 1 | MK011215 | 2 | MH996390 MH996157 |
| *Oenone fulgida* (Savigny in Lamarck, 1818) | 144,726 | 2 | MK011216 MH995737 | 4 | MH996158-60 MH996391 |
| *Olavius (Coralliodriloides) loisae* Erséus, 1984 | 127,965 | 3 | MH995738-39 MK011219 | 2 | MH996162-63 |
| *Olavius albidus* (Jamieson, 1977) | 190,000 | 2 | MK011217-18 | 2 | MH996392 MH996161 |
| Oligochaeta gen. sp. (unidentified Crassiclitellata - Place Kabary 2) | 146,018 | 4 | MH995774-77 | 5 | MH996214-18 |
| *Ophelina acuminata* Örsted, 1843 | 81,846 | 2 | MH995740-41 | 5 | MH996164-67 MH996393 |
| *Ophryotrocha globopalpata* Blake & Hilbig, 1990 | 129,45 |  |  |  |  |
| *Osedax mucofloris* Glover, Kallstrom, Smith & Dahlgren, 2005 | 40,905 | 3 | MH995742-44 | 2 | MH996168 MH996394 |
| *Osedax* sp. | 40,815 |  |  |  |  |
| *Owenia fusiformis* Delle Chiaje, 1844 | 106,476 | 1 | MK011220 | 3 | MH996169-70 MH996395 |
| *Oxydromus pugettensis* (Johnson, 1901) | 92,341 |  |  |  |  |
| *Oxydromus* sp. | 119,100 |  |  |  |  |
| *Palola* sp*.* | 211,279 | 1 | MH995745 | 5 | MH996171-75 |
| *Parachilota* sp. | 72,933 | 2 | MH995746-47 | 3 | MH996176-78 |
| *Paralvinella palmiformis* Desbruyères & Laubier, 1986 | 85,363 | 3 | MK011221-22 MH995748 | 2 | MH996396-97 |
| *Paramphinome jeffreysii* (McIntosh, 1868) | 165,337 | 5 | MK011223-25 MH995749-50 | 5 | MH996179-81 MH996398-99 |
| *Paranais* sp*.* | 100,443 | 3 | MH995751-52 MK011226 | 2 | MH996182 MH996400 |
| *Parvidrilus meyssonnieri* DesChâtelliers & Martin, 2012 | 108,756 |  |  |  |  |
| *Pectinaria gouldii* (Verrill, 1874) | 81,138 | 5 | MH995755-58 MK011228 | 7 | MH996187-93 |
| *Perinereis* sp. | 129,117 | 2 | MH995763-64 | 2 | MH996203-04 |
| *Phagodrilus* sp*.* | 80,487 | 5 | MH995765-69 | 2 | MH996205-06 |
| *Phascolosoma agassizii* Keferstein, 1866 | 87,403 |  |  |  |  |
| *Pherecardia striata* (Kinberg, 1857) | 216,722 | 2 | MH995770 MK011229 | 3 | MH996207-09 |
| *Pherusa plumosa* (Müller, 1776) | 170,126 | 1 | MK011230 | 1 | MH996403 |
| Phreodrilidae gen. sp. 1 | 83,059 | 2 | MH995772-73 | 2 | MH996212-13 |
| Phreodrilidae gen. sp. 2 | 128,500 |  |  |  |  |
| Phreodrilidae gen. sp. 3 | 71,564 |  |  |  |  |
| *Phyllochaetopterus prolifica* Potts, 1914 | 193,836 |  |  |  |  |
| *Piscicola geometra* (Linnaeus, 1761) | 90,720 |  |  |  |  |
| *Pista macrolobata* Hessle, 1917 | 126,764 |  |  |  |  |
| *Poeobius meseres* Heath, 1930 | 70,078 | 3 | MH995760-62 | 6 | MH996197-202 |
| *Polygordius* sp. | 171,704 |  |  |  |  |
| *Pontodrilus litoralis* (Grube, 1855) | 90,268 | 6 | MH995780-83 MK011231-32 | 6 | MH996224-28 MH996404 |
| *Pontoscolex corethrurus* (Muller, 1857) | 193,754 |  |  |  |  |
| *Praxillella pacifica* Berkley, 1929 | 150,768 | 3 | MK011233-34 MH995784 | 5 | MH996229-33 |
| *Prionospio dubia* Day, 1961 | 119,949 | 3 | MH995785-86 MK011235 | 6 | MH996234-39 |
| *Propappus volki* Michaelsen, 1916 | 131,574 | 2 | MH995789-90 | 3 | MH996243-45 |
| *Proscoloplos cygnochaetus* Day, 1954 | 231,508 | 2 | MH995791 MK011236 | 4 | MH996246-48 MH996406 |
| *Protodriloides chaetifer* (Remane, 1926) | 102,702 | 2 | MH995865-66 |  |  |
| *Protomystides* sp. | 109,666 |  |  |  |  |
| *Pseudonereis variegata* (Grube, 1857) | 138,332 | 1 | MH995792 | 1 | MH996407 |
| *Randiella* sp*.* | 151,934 | 11 | MH995793-99 MK011237-39 MK011255 | 4 | MH996249-52 |
| *Rhinodrilus priollii* Righi, 1967 | 87,158 | 2 | MH995800-01 | 3 | MH996253-55 |
| *Rhyacodrilus falciformis* Bretscher, 1901 | 140,129 | 4 | MH995802-03 MK011256 MK011240 | 3 | MH996256-58 |
| *Sabaco elongatus* (Verrill, 1873) | 84,082 | 1 | MH995804 | 2 | MH996259-60 |
| *Saccocirrus papillocercus* Bobretzky, 1872 | 44,209 |  |  |  |  |
| *Scalibregma inflatum* Rathke, 1843 | 126,107 | 5 | MH995807-10 MK011241 | 8 | MH996268-74 MH996408 |
| *Scherotheca* sp. | 113,157 |  |  |  |  |
| *Schizobranchia insignis* Bush, 1905 | 102,002 |  |  |  |  |
| *Sclerolinum brattstromi* Webb, 1964 | 149,694 | 2 | MH995811-12 | 8 | MH996275-80 MH996409-10 |
| *Scolelepis squamata* (Müller, 1806) | 147,343 | 4 | MH995813-16 | 1 | MH996411 |
| *Serpula vermicularis* Linnaeus, 1767 | 151,097 | 2 | MH995818-19 | 5 | MH996283-87 |
| *Siboglinum ekmani* Jägersten, 1956 | 270,658 | 1 | MH995820 | 7 | MH996288-93 MH996412 |
| *Siboglinum fiordicum* Webb, 1963 | 75,226 |  |  |  |  |
| *Sigalion* sp. | 183,997 |  |  |  |  |
| *Sparganophilus* sp. | 123,905 | 5 | MH995821-24 MK011242 | 4 | MH996413-15 MH996294 |
| *Sphaerodorum papillifer* Moore, 1909 | 52,411 |  |  |  |  |
| *Spirobrachia* sp. | 15,375 |  |  |  |  |
| *Spirobrachia* sp. (from Kodiak seep) | 531 |  |  |  |  |
| *Spirobranchus kraussii* (Baird, 1865) | 167,761 | 2 | MH995778-79 | 5 | MH996219-23 |
| *Sternaspis scutata* (Ranzani, 1817) | 115,096 | 3 | MK011245-47 | 9 | MH996419-21 MH996303-08 |
| *Sternaspis* sp*.* | 120,636 | 2 | MK011244 MH995827 | 6 | MH996299-302 MH996417-18 |
| *Sthenelanella uniformis* Moore, 1910 | 103,633 |  |  |  |  |
| *Streblosoma hartmanae* Kritzler, 1971 | 108,080 |  |  |  |  |
| *Stygocapitella subterranea* 1 Knöllner, 1934 | 48,107 |  |  |  |  |
| *Stygocapitella subterranea* 2 Knöllner, 1934 | 74,556 | 2 | MH995828-29 |  |  |
| *Stylodrilus heringianus* Claparède, 1862 | 239,935 | 4 | MH995830-33 | 4 | MH996309-12 |
| *Syllis* cf*. hyalina* Grube, 1863 | 106,283 | 4 | MH995834-37 | 9 | MH996313-21 |
| *Synelmis* sp. | 130,373 |  |  |  |  |
| *Terebellides stroemii* Sars, 1835 | 169,760 | 4 | MH995838-40 MK011248 | 12 | MH996322-32 MH996422 |
| *Terebellobranchia hiata* (Treadwell, 1931) | 35,134 |  |  |  |  |
| *Thalassodrilides* sp*.* | 105,393 | 1 | MH995841 | 5 | MH996333-36 MH996423 |
| *Tharyx kirkegaardi* Blake, 1991 | 114,157 | 4 | MH995842-44 MK011249 | 3 | MH996337-38 MH996424 |
| *Thelepus crispus* Johnson, 1901 | 67,478 | 1 | MH995845 | 1 | MH996339 |
| *Themiste pyroides* (Chamberlin, 1919) | 88,157 |  |  |  |  |
| *Theromyzon tessulatum* (O.F. Müller, 1774) | 63,059 |  |  |  |  |
| *Thysanocardia nigra* (Ikeda, 1904) | 58,011 | 1 | MH995846 | 3 | MH996340-42 |
| *Timarete punctata* (Grube, 1859) | 80,306 | 2 | MH995847-48 | 2 | MH996343-44 |
| *Tomopteris* sp. | 66,655 | 2 | MH995849-50 | 3 | MH996345-47 |
| *Travisia brevis* Moore, 1923 | 69,827 | 1 | MK011250 | 3 | MH996352-53 MH996425 |
| *Triannulata magna* Goodnight, 1940 | 57,647 |  |  |  |  |
| *Tritogenia sulcata* Kinberg, 1867 | 153,752 |  |  |  |  |
| Trochochaetidae gen. sp. | 94,715 |  |  |  |  |
| *Troglodrilus jugeti* Achurra, Châtelliers & Rodriguez, 2012 | 157,399 | 2 | MH995852-53 | 1 | MH996354 |
| *Trypanosyllis* sp. | 167,501 |  |  |  |  |
| *Urobenus* sp. | 55,709 |  |  |  |  |
| *Vignysa popi* Bouché, 1970 | 93,260 | 2 | MH995854-55 | 1 | MH996355 |
| *Xironogiton victoriensis* Gelder and Hall, 1990 | 55,289 | 1 | MK011251 |  |  |
