# Supplementary material for: Newly Discovered Occurrences and Gene Tree of the Extracellular Globins and Linker Chains from the Giant Hexagonal Bilayer Hemoglobin in Metazoans
Source: Genome Biol Evol. 2019 Jan 21;11(3):597–612. doi: 10.1093/gbe/evz012 (PMC6400237; doi:10.1093/gbe/evz012)
Supplement: Supplementary Data [file evz012_supp.zip › Supplementary_file2.docx]

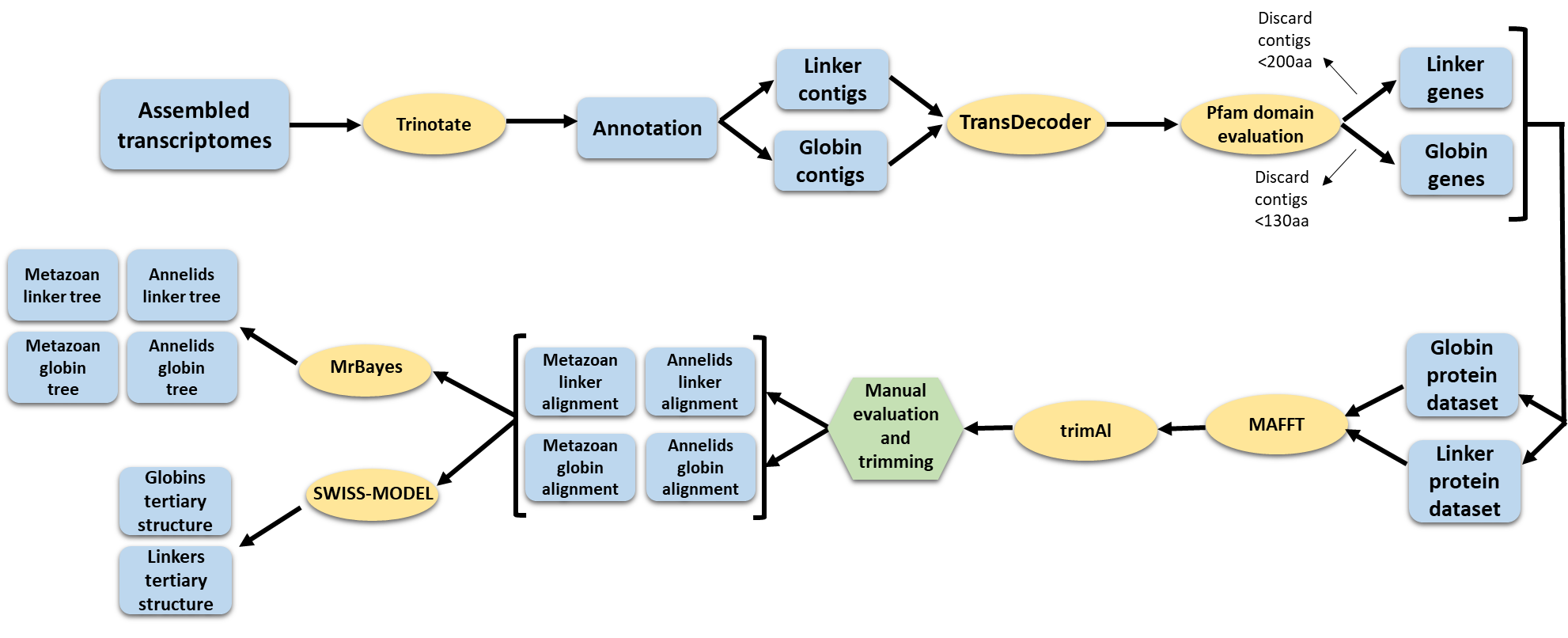


**Supplementary file 2** - Flow chart of our bioinformatics pipeline modified from Costa-Paiva et al. (2017). Rounded blue rectangles represent input/output files, yellow ovals represent software or scripts, and the green hexagon represents a step which involving manual evaluation.
