# Supplementary material for: Newly Discovered Occurrences and Gene Tree of the Extracellular Globins and Linker Chains from the Giant Hexagonal Bilayer Hemoglobin in Metazoans
Source: Genome Biol Evol. 2019 Jan 21;11(3):597–612. doi: 10.1093/gbe/evz012 (PMC6400237; doi:10.1093/gbe/evz012)
Supplement: Supplementary Data [file evz012_supp.zip › Supplementary_file5.docx]

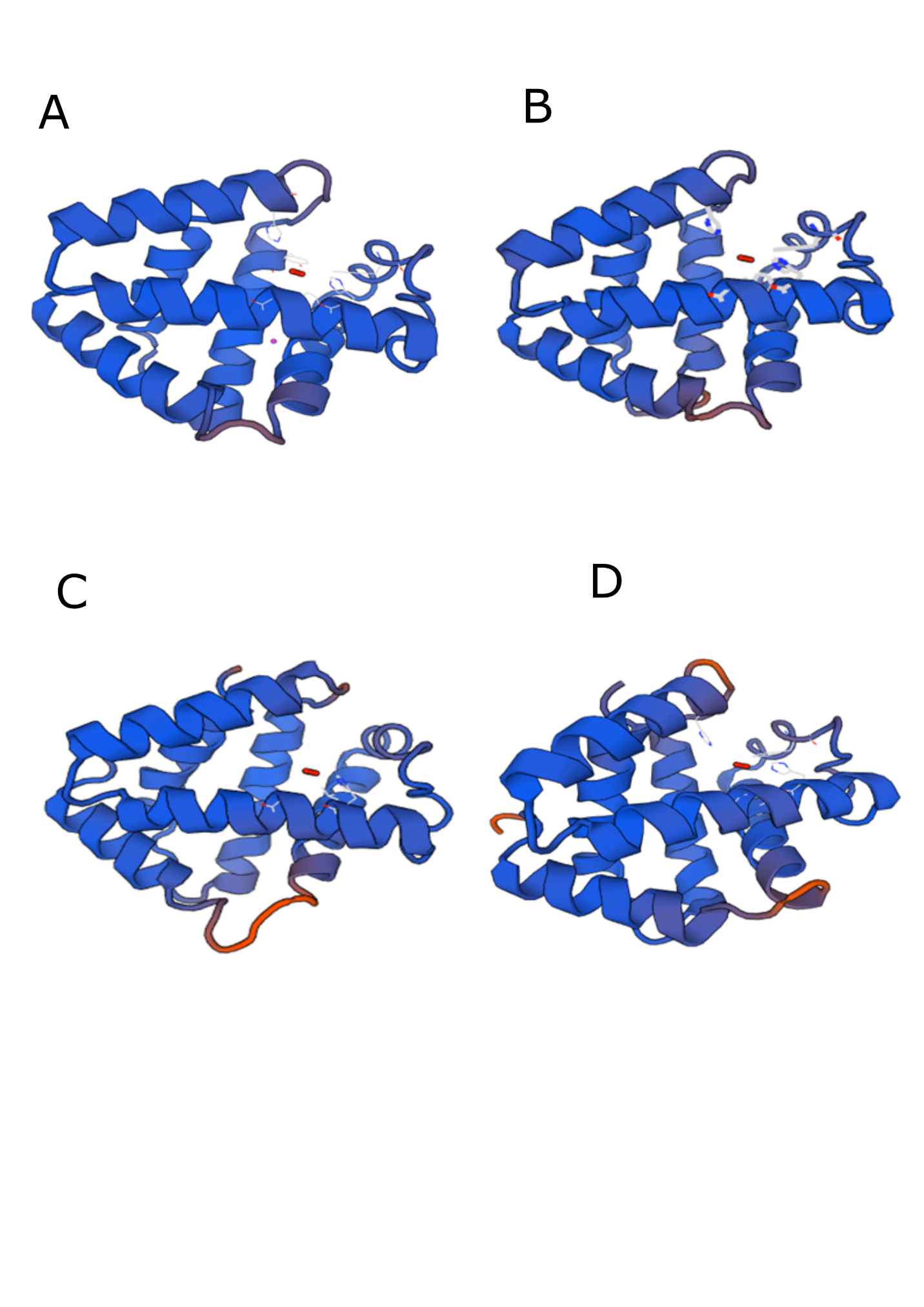


**Supplementary file 5** - Tertiary structure of each one of the extracellular globin subtypes inferred using SWISS-MODEL (Arnold et al., 2006; Kiefer et al., 2009) The red dot in each figure represents di-oxygen bound to the globin. These models suggest that all sequences have a putative respiratory function and also showed the high similarity among their tertiary structure. (A) Stereobalanus canadensis – globin chain A1 (Hemichordata); (B) Cephalodiscus gracilis – globin chain A2 (Hemichordata); (C) Kruppomenia borealis – globin chain B1 (Mollusca); (D) Novocrania anomala – globin chain B2 (Brachiopoda).
