# Supplementary material for: Newly Discovered Occurrences and Gene Tree of the Extracellular Globins and Linker Chains from the Giant Hexagonal Bilayer Hemoglobin in Metazoans
Source: Genome Biol Evol. 2019 Jan 21;11(3):597–612. doi: 10.1093/gbe/evz012 (PMC6400237; doi:10.1093/gbe/evz012)
Supplement: Supplementary Data [file evz012_supp.zip › Supplementary_file6.docx]

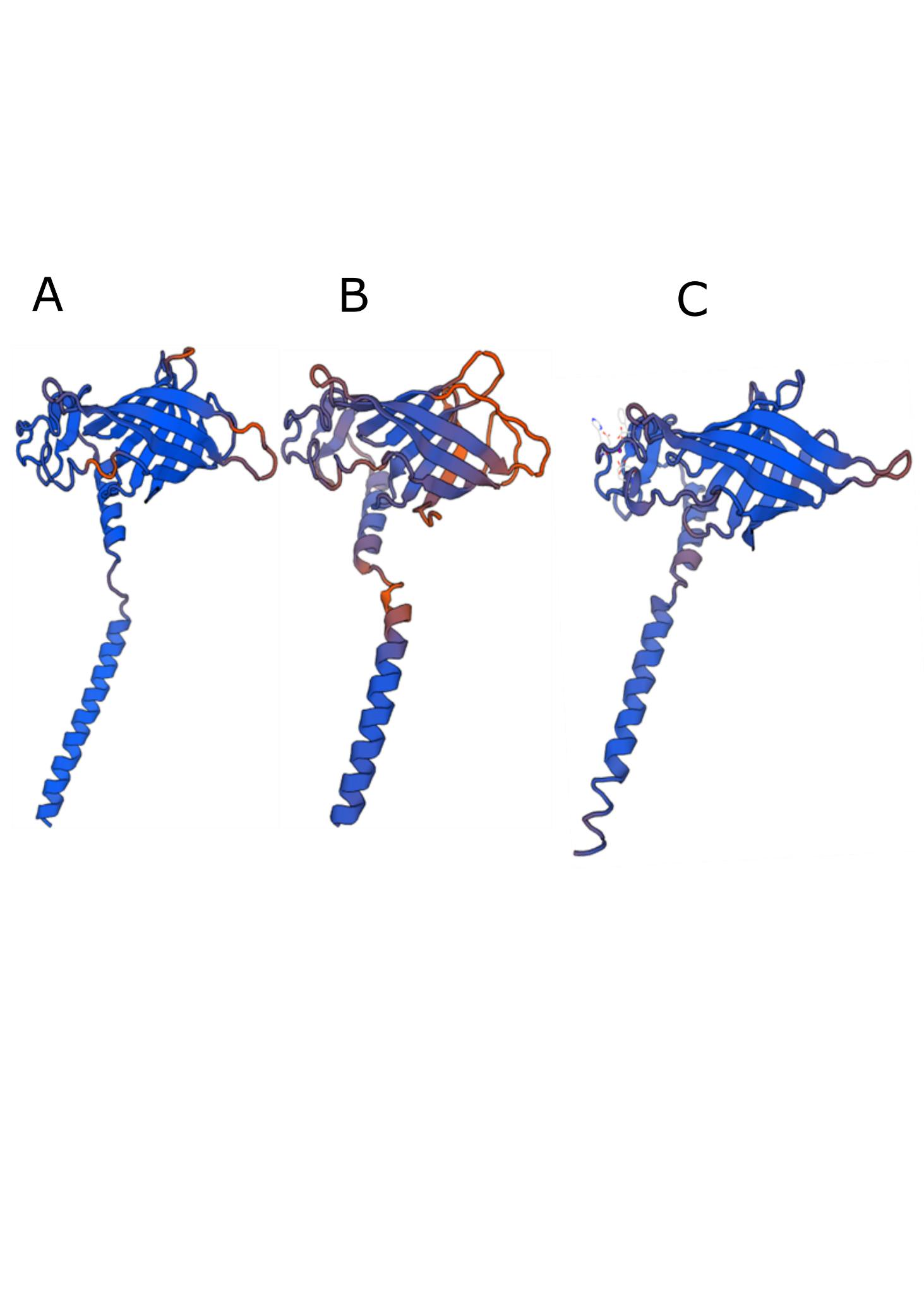


**Supplementary file 6** – Tertiary structure of each one of the three linker chains inferred using SWISS-MODEL (Arnold et al., 2006; Kiefer et al., 2009). There appears to be high similarity among the tertiary structures. (A) Hemithiris psittacea – linker chain L1 (Brachiopoda); (B) Labidiaster annulatus – linker chain L2 (Echinodermata); (C) Priapulus sp. – linker chain L3 (Priapulida).
