# Supplementary material for: Newly Discovered Occurrences and Gene Tree of the Extracellular Globins and Linker Chains from the Giant Hexagonal Bilayer Hemoglobin in Metazoans
Source: Genome Biol Evol. 2019 Jan 21;11(3):597–612. doi: 10.1093/gbe/evz012 (PMC6400237; doi:10.1093/gbe/evz012)
Supplement: Supplementary Data [file evz012_supp.zip › Supplementary_file7.docx]

**Supplementary file 7** – List of genomes from GenBank analyzed and their respective assembly and ID numbers. No extracellular globin or linkers genes were found in any of the genomes.

| **Taxon** | **Genbank assembly** | **ID** | **Globin genes number** | **Linker genes number** |
| --- | --- | --- | --- | --- |
| PORIFERA |  |  |  |  |
| *Amphimedon queenslandica* | [GCA_000090795.1](https://www.ncbi.nlm.nih.gov/assembly/293608) | 2698 | -- | -- |
| CNIDARIA |  |  |  |  |
| *Hydra vulgaris* | [GCA_000004095.1](https://www.ncbi.nlm.nih.gov/assembly/GCA_000004095.1) | 12836 | -- | -- |
| *Nematostella vectensis* | GCA_000209225.1 | 230 | -- | -- |
| CTENOPHORA |  |  |  |  |
| *Pleurobrachia bachei* | [GCA_000695325.1](https://www.ncbi.nlm.nih.gov/assembly/180401) | 12376 | -- | -- |
| CHORDATA |  |  |  |  |
| *Cyprinus carpio* | [GCA_000951615.2](https://www.ncbi.nlm.nih.gov/assembly/GCA_000951615.2) | 10839 | -- | -- |
| *Mus musculus* | [GCA_000001635.8](https://www.ncbi.nlm.nih.gov/assembly/GCA_000001635.8) | 52 | -- | -- |
| PLACOZOA |  |  |  |  |
| *Trichoplax adhaerens* | [GCA_000150275.1](https://www.ncbi.nlm.nih.gov/assembly/173428) | 354 | -- | -- |
| NEMATODA |  |  |  |  |
| *Caenorhabditis elegans* | GCA_000002985.3 | 41 | -- | -- |
| *Pristionchus pacificus* | [GCA_000180635.3](https://www.ncbi.nlm.nih.gov/assembly/1304971) | 246 | -- | -- |
| TARDIGRADA |  |  |  |  |
| *Ramazzottius varieornatus* | GCA_001949185.1 | 46574 | -- | -- |
| ARTHROPODA |  |  |  |  |
| *Apis mellifera* | [GCA_003254395.2](https://www.ncbi.nlm.nih.gov/assembly/GCA_003254395.2) | 48 | -- | -- |
| *Penaeus japonicus* | [GCA_002291165.1](https://www.ncbi.nlm.nih.gov/assembly/1192181) | 24063 | -- | -- |
| *Sarcoptes scabiei* | [GCA_000828355.1](https://www.ncbi.nlm.nih.gov/assembly/251951) | 36095 | -- | -- |
