# Supplementary material for: Newly Discovered Occurrences and Gene Tree of the Extracellular Globins and Linker Chains from the Giant Hexagonal Bilayer Hemoglobin in Metazoans
Source: Genome Biol Evol. 2019 Jan 21;11(3):597–612. doi: 10.1093/gbe/evz012 (PMC6400237; doi:10.1093/gbe/evz012)
Supplement: Supplementary Data [file evz012_supp.zip › Supplementary_file8.docx]

**Supplementary file 7 –** Linker genes accession numbers for each species.

| **Taxon** | **Linker accession number** |
| --- | --- |
| **Echinodermata** |  |
| *Astrotoma agassizii* | MH995560 |
| *Labidiaster annulatus* 1 | MH995678 |
| *Labidiaster annulatus* 2 | MH995679 |
| **Hemichordata** |  |
| *Balanoglossus aurantiaca* | MH995569 |
| *Cephalodiscus gracilis* | MH995586 |
| *Stereobalanus canadensis* 1 | MH995825 |
| *Stereobalanus canadensis* 2 | MH995826 |
| Torquaratoridae gen. sp. 1 - 1 | MH995851 |
| **Annelida** |  |
| *Abarenicola pacifica* 1 | MH995534 |
| *Abarenicola pacifica* 2 | MK011176 |
| *Aeolosoma* sp. 1 | MH995536 |
| *Aeolosoma* sp. 2 | MH995537 |
| *Aeolosoma* sp. 3 | MH995538 |
| *Aeolosoma* sp. 4 | MH995539 |
| *Aglaophamus verrilli* 1 | MK011177 |
| *Aglaophamus verrilli* 2 | MH995540 |
| *Alitta succinea* 1 | MH995729 |
| *Alitta succinea* 2 | MH995730 |
| *Alitta succinea* 3 | MK011213 |
| *Amynthas* sp. 1 | MH995554 |
| *Amynthas* sp. 2 | MK011182 |
| *Amynthas* sp. 3 | MK011183 |
| *Amynthas* sp. 4 | MH995555 |
| *Amynthas* sp. 5 | MH995556 |
| *Amynthas* sp. 6 | MH995557 |
| *Amynthas* sp. 7 | MH995558 |
| *Amynthas* sp. 8 | MH995559 |
| *Andiorrhinus* sp. 1 | MH995541 |
| *Andiorrhinus* sp. 2 | MH995542 |
| *Andiorrhinus* sp. 3 | MH995543 |
| *Antarctodrilus proboscidea* | MH995544 |
| *Aphelochaeta* sp. 1 | MK011178 |
| *Aphelochaeta* sp. 2 | MK011179 |
| *Aphelochaeta* sp. 3 | MH995545 |
| *Aphelochaeta* sp. 4 | MH995546 |
| *Aphelochaeta* sp. 5 | MH995547 |
| *Aphrodita japonica* 1 | MH995548 |
| *Aphrodita japonica* 2 | MH995549 |
| *Aphrodita japonica* 3 | MH995550 |
| *Aphrodita japonica* 4 | MH995551 |
| *Aphrodita japonica* 5 | MH995552 |
| *Aphrodita japonica* 6 | MK011180 |
| *Arabella* sp*.* | MH995553 |
| *Aricidea quadrilobata* | MH995864 |
| *Armandia* sp*.* | MK011181 |
| *Auchenoplax crinita* 1 | MH995561 |
| *Auchenoplax crinita* 2 | MH995562 |
| *Auchenoplax crinita* 3 | MH995563 |
| *Auchenoplax crinita* 4 | MK011184 |
| *Auchenoplax crinita* 5 | MH995564 |
| *Aulodrilus japonicus* 1 | MH995565 |
| *Aulodrilus japonicus* 2 | MH995566 |
| *Aulodrilus japonicus* 3 | MK011185 |
| *Aulodrilus japonicus* 4 | MK011186 |
| *Axiothella rubrocincta* 1 | MH995567 |
| *Axiothella rubrocincta* 2 | MH995568 |
| *Bathydrilus rohdei* 1 | MH995570 |
| *Bathydrilus rohdei* 2 | MH995571 |
| *Bathydrilus rohdei* 3 | MH995572 |
| *Bathydrilus rohdei* 4 | MH995573 |
| *Bispira pacifica* 1 | MH995805 |
| *Bispira pacifica* 2 | MH995806 |
| *Boccardia proboscidea* 1 | MH995574 |
| *Boccardia proboscidea* 2 | MK011187 |
| *Bothrioneurum vejdovskyanum* | MH995575 |
| *Branchiobdella kobayashii* 1 | MH995576 |
| *Branchiobdella kobayashii* 2 | MH995577 |
| *Branchiobdella parasita* 1 | MH995578 |
| *Branchiobdella parasita* 2 | MH995579 |
| *Cambarincola gracilis* 1 | MH995580 |
| *Cambarincola gracilis* 2 | MH995581 |
| *Cambarincola holti* 1 | MH995582 |
| *Cambarincola holti* 2 | MH995583 |
| *Cambarincola holti* 3 | MH995584 |
| *Capilloventer* sp. 1 | MH995585 |
| *Capilloventer* sp. 2 | MK011188 |
| *Chaetogaster diaphanus* 1 | MH995587 |
| *Chaetogaster diaphanus* 2 | MH995588 |
| *Chaetozone* sp. 1 | MH995589 |
| *Chaetozone* sp. 2 | MK011189 |
| *Chaetozone* sp. 3 | MH995590 |
| *Chaetozone* sp. 4 | MH995591 |
| *Chloeia pinnata* 1 | MK011190 |
| *Chloeia pinnata* 2 | MK011191 |
| *Cirratulus spectabilis* 1 | MK011192 |
| *Cirratulus spectabilis* 2 | MH995592 |
| *Cirrodrilus suzukii* 1 | MH995593 |
| *Cirrodrilus suzukii* 2 | MK011193 |
| *Cirrodrilus suzukii* 3 | MH995594 |
| *Clymenella torquata* 1 | MH995595 |
| *Clymenella torquata* 2 | MH995596 |
| *Cossura longocirrata* 1 | MH995597 |
| *Cossura longocirrata* 2 | MH995598 |
| *Cossura longocirrata* 3 | MH995599 |
| *Cossura longocirrata* 4 | MK011194 |
| *Cossura longocirrata* 5 | MH995600 |
| *Crucigera zygophora* | MH995601 |
| *Delaya leruthi* 1 | MH995602 |
| *Delaya leruthi* 2 | MH995603 |
| *Delaya leruthi* 3 | MH995604 |
| *Dichogaster* green tree worm 1 | MH995605 |
| *Dichogaster* green tree worm 2 | MH995606 |
| *Dichogaster* green tree worm 3 | MH995607 |
| *Dichogaster* green tree worm 4 | MH995608 |
| *Dichogaster guadeloupensis* | MH995609 |
| *Dichogaster saliens* | MH995610 |
| *Diplocardia* sp. 1 | MH995611 |
| *Diplocardia* sp. 2 | MK011195 |
| *Diplocardia* sp. 3 | MH995612 |
| *Diplocardia* sp. 4 | MH995613 |
| *Dodecaceria pulchra* 1 | MH995614 |
| *Dodecaceria pulchra* 2 | MH995615 |
| *Dodecaceria pulchra* 3 | MH995616 |
| *Dorydrilus michaelseni* 1 | MH995617 |
| *Dorydrilus michaelseni* 2 | MH995618 |
| *Drawida* sp. 1 | MH995619 |
| *Drawida* sp. 2 | MH995620 |
| *Drilocrius* sp*.* 1 | MH995621 |
| *Drilocrius* sp*.* 2 | MH995622 |
| *Drilocrius* sp*.* 3 | MH995623 |
| *Drilocrius* sp*.* 4 | MK011196 |
| Echiura gen. sp. green | MH995624 |
| *Eisenia* sp*.* 1 | MH995856 |
| *Eisenia* sp*.* 2 | MH995857 |
| *Eisenia* sp*.* 3 | MH995858 |
| *Eisenia* sp*.* 4 | MH995859 |
| *Enchytraeus albidus* 1 | MH995626 |
| *Enchytraeus albidus* 2 | MH995627 |
| *Erpobdella octoculata* | MH995628 |
| *Eunice norvegica* 1 | MH995629 |
| *Eunice norvegica* 2 | MH995630 |
| *Eunice pennata* | MH995631 |
| *Flabegraviera mundata* | MH995632 |
| *Galathowenia oculata* 1 | MH995633 |
| *Galathowenia oculata* 2 | MH995634 |
| *Galathowenia oculata* 3 | MH995635 |
| *Galathowenia oculata* 4 | MH995636 |
| *Galeolaria caespitosa* 1 | MH995637 |
| *Galeolaria caespitosa* 2 | MH995638 |
| *Gatesona chaetophora* | MH995639 |
| *Geogenia benhami* 1 | MH995640 |
| *Geogenia benhami* 2 | MH995641 |
| *Glossodrilus* sp*.* 1 | MK011197 |
| *Glossodrilus* sp*.* 2 | MH995642 |
| *Glycera dibranchiata* | MH995643 |
| *Glyptonotobdella antarctica* | MK011198 |
| *Goniada brunnea* | MH995644 |
| *Grania* sp*.* 1 | MH995860 |
| *Grania* sp*.* 2 | MH995861 |
| *Grania* sp*.* 3 | MH995862 |
| *Grania* sp*.* 4 | MH995863 |
| *Guaranidrilus* sp*.* | MH995645 |
| *Halosydna brevisetosa* 1 | MH995646 |
| *Halosydna brevisetosa* 2 | MH995647 |
| *Halosydna brevisetosa* 3 | MH995648 |
| Haplotaxidae gen. sp. 1 - 1 | MH995649 |
| Haplotaxidae gen. sp. 1 - 2 | MH995650 |
| *Haplotaxis gordioides* | MH995651 |
| *Haplotaxis* sp. 1 | MH995652 |
| *Haplotaxis* sp. 2 | MH995653 |
| *Hemigastrodrilus monicae* 1 | MH995654 |
| *Hemigastrodrilus monicae* 2 | MH995655 |
| *Hemigastrodrilus monicae* 3 | MH995656 |
| *Hemigastrodrilus monicae* 4 | MK011199 |
| *Hemigastrodrilus monicae* 5 | MH995657 |
| *Hermodice carunculata* | MK011201 |
| *Heronidrilus* sp. 1 | MK011202 |
| *Heronidrilus* sp. 2 | MH995662 |
| *Heronidrilus* sp. 3 | MK011203 |
| *Hesionides* sp*.* | MH995663 |
| *Heterodrilus* sp. 1 - 1 | MK011204 |
| *Heterodrilus* sp. 1 - 2 | MK011252 |
| *Heterodrilus* sp. 1 - 3 | MH995664 |
| *Heterodrilus* sp. 1 - 4 | MK011205 |
| *Hrabeiella periglandulata* 1 | MH995665 |
| *Hrabeiella periglandulata* 2 | MH995666 |
| *Idanthyrsus* sp. 1 | MH995668 |
| *Idanthyrsus* sp. 2 | MH995669 |
| *Kincaidiana* sp. 1 | MH995670 |
| *Kincaidiana* sp. 2 | MH995671 |
| *Kincaidiana* sp. 3 | MH995672 |
| *Kincaidiana* sp. 4 | MH995673 |
| *Komarekiona eatoni* 1 | MH995674 |
| *Komarekiona eatoni* 2 | MH995675 |
| *Komarekiona eatoni* 3 | MH995676 |
| *Lamellibrachia luymesi* 1 | MH995680 |
| *Lamellibrachia luymesi* 2 | MH995681 |
| *Laonice* sp. | MH995682 |
| *Leitoscoloplos robustus* 1 | MH995683 |
| *Leitoscoloplos robustus* 2 | MH995684 |
| *Leitoscoloplos robustus* 3 | MH995685 |
| *Lepidonotus semitectus* 1 | MK011206 |
| *Lepidonotus semitectus* 2 | MH995686 |
| *Lepidonotus semitectus* 3 | MH995687 |
| *Limnodriloides* sp*.* 1 | MH995689 |
| *Limnodriloides* sp*.* 2 | MH995690 |
| *Limnodriloides* sp*.* 3 | MH995691 |
| *Limnodriloides* sp*.* 4 | MH995692 |
| *Lumbriculus variegatus* 1 | MH995693 |
| *Lumbriculus variegatus* 2 | MH995694 |
| *Lumbriculus variegatus* 3 | MK011253 |
| *Lumbriculus variegatus* 4 | MH995695 |
| *Lumbrineris crassicephala* 1 | MH995696 |
| *Lumbrineris crassicephala* 2 | MK011207 |
| *Lumbrineris crassicephala* 3 | MH995697 |
| *Lumbrineris crassicephala* 4 | MH995698 |
| *Lumbrineris crassicephala* 5 | MH995699 |
| *Lumbrineris crassicephala* 6 | MH995700 |
| *Lumbrineris perkinsi* | MH995701 |
| *Lutodrilus* sp. 1 | MH995702 |
| *Lutodrilus* sp. 2 | MH995703 |
| *Lutodrilus* sp. 3 | MH995704 |
| *Macrochaeta* sp. 1 | MK011208 |
| *Macrochaeta* sp. 2 | MH995705 |
| *Magelona berkeleyi* | MH995706 |
| *Marphysa sanguinea* | MH995707 |
| *Melinna maculata* 1 | MH995708 |
| *Melinna maculata* 2 | MH995709 |
| *Melinna maculata* 3 | MK011209 |
| *Melinna maculata* 4 | MK011210 |
| *Melinna maculata* 5 | MH995710 |
| *Mesenchytraeus pedatus* 1 | MH995711 |
| *Mesenchytraeus pedatus* 2 | MH995712 |
| *Mesenchytraeus solifugus* DARK | MH995713 |
| *Mesenchytraeus solifugus* LIGHT | MH995714 |
| *Mesenchytraeus* sp. 1 | MH995715 |
| *Mesenchytraeus* sp. 2 | MH995716 |
| Microchaetidae gen. sp. 1 - 1 | MH995717 |
| Microchaetidae gen. sp. 1 - 2 | MK011211 |
| *Microchaetus* sp. | MH995718 |
| *Microphthalmus similis* | MH995719 |
| *Myxicola infundibulum* 1 | MH995720 |
| *Myxicola infundibulum* 2 | MH995721 |
| *Myxicola infundibulum* 3 | MH995722 |
| *Myxicola infundibulum* 4 | MH995723 |
| *Naineris laevigata* 1 | MH995724 |
| *Naineris laevigata* 2 | MH995725 |
| *Naineris laevigata* 3 | MH995726 |
| *Neosabellaria cementarium* | MK011254 |
| *Nephtys incisa* 1 | MH995727 |
| *Nephtys incisa* 2 | MH995728 |
| *Nephtys incisa* 3 | MK011212 |
| *Nicolea macrobranchia* 1 | MH995731 |
| *Nicolea macrobranchia* 2 | MH995732 |
| *Nicomache venticola* 1 | MH995733 |
| *Nicomache venticola* 2 | MH995734 |
| *Ninoe nigripes* 1 | MH995735 |
| *Ninoe nigripes* 2 | MK011214 |
| *Odontosyllis gibba* | MK011215 |
| *Oenone fulgida* 1 | MK011216 |
| *Oenone fulgida* 2 | MH995737 |
| *Olavius (Coralliodriloides) loisae* 1 | MH995738 |
| *Olavius (Coralliodriloides) loisae* 2 | MH995739 |
| *Olavius (Coralliodriloides) loisae* 3 | MK011219 |
| *Olavius albidus* 1 | MK011217 |
| *Olavius albidus* 2 | MK011218 |
| Oligochaeta gen. sp. (unidentified Crassiclitellata - Place Kabary 2) - 1 | MH995774 |
| Oligochaeta gen. sp. (unidentified Crassiclitellata - Place Kabary 2) - 2 | MH995775 |
| Oligochaeta gen. sp. (unidentified Crassiclitellata - Place Kabary 2) - 3 | MH995776 |
| Oligochaeta gen. sp. (unidentified Crassiclitellata - Place Kabary 2) - 4 | MH995777 |
| *Ophelina acuminata* 1 | MH995740 |
| *Ophelina acuminata* 2 | MH995741 |
| *Osedax mucofloris* 1 | MH995742 |
| *Osedax mucofloris* 2 | MH995743 |
| *Osedax mucofloris* 3 | MH995744 |
| *Owenia fusiformis* | MK011220 |
| *Palola* sp*.* | MH995745 |
| *Parachilota* sp. 1 | MH995746 |
| *Parachilota* sp. 2 | MH995747 |
| *Paralvinella palmiformis* 1 | MK011221 |
| *Paralvinella palmiformis* 2 | MH995748 |
| *Paralvinella palmiformis* 3 | MK011222 |
| *Paramphinome jeffreysii* 1 | MK011223 |
| *Paramphinome jeffreysii* 2 | MK011224 |
| *Paramphinome jeffreysii* 3 | MH995749 |
| *Paramphinome jeffreysii* 4 | MH995750 |
| *Paramphinome jeffreysii* 5 | MK011225 |
| *Paranais* sp*.* 1 | MH995751 |
| *Paranais* sp*.* 2 | MH995752 |
| *Paranais* sp*.* 3 | MK011226 |
| *Pectinaria gouldii* 1 | MH995755 |
| *Pectinaria gouldii* 2 | MH995756 |
| *Pectinaria gouldii* 3 | MH995757 |
| *Pectinaria gouldii* 4 | MH995758 |
| *Pectinaria gouldii* 5 | MK011228 |
| *Perinereis* sp. 1 | MH995763 |
| *Perinereis* sp. 2 | MH995764 |
| *Phagodrilus* sp*.* 1 | MH995765 |
| *Phagodrilus* sp*.* 2 | MH995766 |
| *Phagodrilus* sp*.* 3 | MH995767 |
| *Phagodrilus* sp*.* 4 | MH995768 |
| *Phagodrilus* sp*.* 5 | MH995769 |
| *Pherecardia striata* 1 | MH995770 |
| *Pherecardia striata* 2 | MK011229 |
| *Pherusa plumosa* | MK011230 |
| Phreodrilidae gen. sp. 1 - 1 | MH995772 |
| Phreodrilidae gen. sp. 1 - 2 | MH995773 |
| *Poeobius meseres* 1 | MH995760 |
| *Poeobius meseres* 2 | MH995761 |
| *Poeobius meseres* 3 | MH995762 |
| *Pontodrilus litoralis* 1 | MH995780 |
| *Pontodrilus litoralis* 2 | MK011231 |
| *Pontodrilus litoralis* 3 | MH995781 |
| *Pontodrilus litoralis* 4 | MH995782 |
| *Pontodrilus litoralis* 5 | MH995783 |
| *Pontodrilus litoralis* 6 | MK011232 |
| *Praxillella pacifica* 1 | MK011233 |
| *Praxillella pacifica* 2 | MK011234 |
| *Praxillella pacifica* 3 | MH995784 |
| *Prionospio dubia* 1 | MH995785 |
| *Prionospio dubia* 2 | MK011235 |
| *Prionospio dubia* 3 | MH995786 |
| *Propappus volki* 1 | MH995789 |
| *Propappus volki* 2 | MH995790 |
| *Proscoloplos cygnochaetus* 1 | MH995791 |
| *Proscoloplos cygnochaetus* 2 | MK011236 |
| *Protodriloides chaetifer* 1 | MH995865 |
| *Protodriloides chaetifer* 2 | MH995866 |
| *Pseudonereis variegata* | MH995792 |
| *Randiella* sp*.* 1 | MH995793 |
| *Randiella* sp*.* 2 | MK011237 |
| *Randiella* sp*.* 3 | MH995794 |
| *Randiella* sp*.* 4 | MH995795 |
| *Randiella* sp*.* 5 | MK011238 |
| *Randiella* sp*.* 6 | MH995796 |
| *Randiella* sp*.* 7 | MH995797 |
| *Randiella* sp*.* 8 | MH995798 |
| *Randiella* sp*.* 9 | MK011239 |
| *Randiella* sp*.* 10 | MK011255 |
| *Randiella* sp*.* 11 | MH995799 |
| *Rhinodrilus priollii* 1 | MH995800 |
| *Rhinodrilus priollii* 2 | MH995801 |
| *Rhyacodrilus falciformis* 1 | MH995802 |
| *Rhyacodrilus falciformis* 2 | MK011256 |
| *Rhyacodrilus falciformis* 3 | MH995803 |
| *Rhyacodrilus falciformis* 4 | MK011240 |
| *Sabaco elongatus* | MH995804 |
| *Scalibregma inflatum* 1 | MH995807 |
| *Scalibregma inflatum* 2 | MH995808 |
| *Scalibregma inflatum* 3 | MH995809 |
| *Scalibregma inflatum* 4 | MH995810 |
| *Scalibregma inflatum* 5 | MK011241 |
| *Sclerolinum brattstromi* 1 | MH995811 |
| *Sclerolinum brattstromi* 2 | MH995812 |
| *Scolelepis squamata* 1 | MH995813 |
| *Scolelepis squamata* 2 | MH995814 |
| *Scolelepis squamata* 3 | MH995815 |
| *Scolelepis squamata* 4 | MH995816 |
| *Serpula vermicularis* 1 | MH995818 |
| *Serpula vermicularis* 2 | MH995819 |
| *Siboglinum ekmani* | MH995820 |
| *Sparganophilus* sp. 1 | MH995821 |
| *Sparganophilus* sp. 2 | MK011242 |
| *Sparganophilus* sp. 3 | MH995822 |
| *Sparganophilus* sp. 4 | MH995823 |
| *Sparganophilus* sp. 5 | MH995824 |
| *Spirobranchus kraussii* 1 | MH995778 |
| *Spirobranchus kraussii* 2 | MH995779 |
| *Sternaspis scutata* 1 | MK011245 |
| *Sternaspis scutata* 2 | MK011246 |
| *Sternaspis scutata* 3 | MK011247 |
| *Sternaspis* sp*.* 1 | MK011244 |
| *Sternaspis* sp*.* 2 | MH995827 |
| *Stygocapitella subterranea* 2 - 1 | MH995828 |
| *Stygocapitella subterranea* 2 - 2 | MH995829 |
| *Stylodrilus heringianus* 1 | MH995830 |
| *Stylodrilus heringianus* 2 | MH995831 |
| *Stylodrilus heringianus* 3 | MH995832 |
| *Stylodrilus heringianus* 4 | MH995833 |
| *Syllis* cf. *hyalina* 1 | MH995834 |
| *Syllis* cf. *hyalina* 2 | MH995835 |
| *Syllis* cf. *hyalina* 3 | MH995836 |
| *Syllis* cf. *hyalina* 4 | MH995837 |
| *Terebellides stroemii* 1 | MH995838 |
| *Terebellides stroemii* 2 | MH995839 |
| *Terebellides stroemii* 3 | MK011248 |
| *Terebellides stroemii* 4 | MH995840 |
| *Thalassodrilides* sp*.* | MH995841 |
| *Tharyx kirkegaardi* 1 | MH995842 |
| *Tharyx kirkegaardi* 2 | MH995843 |
| *Tharyx kirkegaardi* 3 | MH995844 |
| *Tharyx kirkegaardi* 4 | MK011249 |
| *Thelepus crispus* | MH995845 |
| *Thysanocardia nigra* | MH995846 |
| *Timarete punctata* 1 | MH995847 |
| *Timarete punctata* 2 | MH995848 |
| *Tomopteris* sp. 1 | MH995849 |
| *Tomopteris* sp. 2 | MH995850 |
| *Travisia brevis* | MK011250 |
| *Troglodrilus jugeti* 1 | MH995852 |
| *Troglodrilus jugeti* 2 | MH995853 |
| *Vignysa popi* 1 | MH995854 |
| *Vignysa popi* 2 | MH995855 |
| *Xironogiton victoriensis* | MK011251 |
| **Brachiopoda** |  |
| *Hemithiris psittacea* 1 | MH995658 |
| *Hemithiris psittacea* 2 | MH995659 |
| *Hemithiris psittacea* 3 | MH995660 |
| *Hemithiris psittacea* 4 | MH995661 |
| *Hemithiris psittacea* 5 | MK011200 |
| *Novocrania anomala* | MH995736 |
| **Phoronida** |  |
| *Phoronis psammophila* | MH995771 |
| **Mollusca** |  |
| *Hypomenia* sp*.* | MH995667 |
| *Kruppomenia borealis* | MH995677 |
| *Leptochiton rugatus* | MH995688 |
| *Spathoderma clenchi* | MK011243 |
| **Nemertea** |  |
| *Paranemertes peregrina* 1 | MH995753 |
| *Paranemertes peregrina* 2 | MK011227 |
| *Paranemertes peregrina* 3 | MH995754 |
| **Bryozoa** |  |
| *Pectinatella magnifica* | MH995759 |
| **Platyhelminthes** |  |
| *Acipensericola petersoni* | MH995535 |
| *Elopicola* sp. | MH995625 |
| *Selachohemecus olsoni* | MH995817 |
| **Priapulida** |  |
| *Priapulus* sp*.* 1 | MH995787 |
| *Priapulus* sp*.* 2 | MH995788 |
