# Supplementary material for: Newly Discovered Occurrences and Gene Tree of the Extracellular Globins and Linker Chains from the Giant Hexagonal Bilayer Hemoglobin in Metazoans
Source: Genome Biol Evol. 2019 Jan 21;11(3):597–612. doi: 10.1093/gbe/evz012 (PMC6400237; doi:10.1093/gbe/evz012)
Supplement: Supplementary Data [file evz012_supp.zip › Supplementary_file9.docx]

**Supplementary file 8** – Globin genes accession numbers for each species.

| **Taxon** | **Globin accession number** |
| --- | --- |
| **METAZOA** |  |
| **Echinodermata** |  |
| *Astrotoma agassizii* 1 | MH995909 |
| *Astrotoma agassizii* 2 | MH996362 |
| *Labidiaster annulatus* 1 | MH996061 |
| *Labidiaster annulatus* 2 | MH996062 |
| **Hemichordata** |  |
| *Cephalodiscus gracilis* 1 | MH995925 |
| *Cephalodiscus gracilis* 2 | MH995926 |
| *Stereobalanus canadensis* 1 | MH996296 |
| *Stereobalanus canadensis* 2 | MH996297 |
| *Stereobalanus canadensis* 3 | MH996298 |
| *Stereobalanus canadensis* 4 | MH996416 |
| Torquaratoridae gen. sp. 1 - 1 | MH996348 |
| Torquaratoridae gen. sp. 1 - 2 | MH996349 |
| Torquaratoridae gen. sp. 1 - 3 | MH996350 |
| Torquaratoridae gen. sp. 1 - 4 | MH996351 |
| **Annelida** |  |
| *Abarenicola pacifica* 1 | MH995867 |
| *Abarenicola pacifica* 2 | MH995868 |
| *Aeolosoma* sp. 1 | MH995869 |
| *Aeolosoma* sp. 2 | MH995870 |
| *Aeolosoma* sp. 3 | MH995871 |
| *Aglaophamus verrilli* 1 | MH995872 |
| *Aglaophamus verrilli* 2 | MH995873 |
| *Aglaophamus verrilli* 3 | MH995874 |
| *Aglaophamus verrilli* 4 | MH995875 |
| *Aglaophamus verrilli* 5 | MH995876 |
| *Aglaophamus verrilli* 6 | MH995877 |
| *Alitta succinea* 1 | MH996143 |
| *Alitta succinea* 2 | MH996144 |
| *Alitta succinea* 3 | MH996145 |
| *Alitta succinea* 4 | MH996146 |
| *Alitta succinea* 5 | MH996147 |
| *Amynthas* sp. 1 | MH995897 |
| *Amynthas* sp. 2 | MH995898 |
| *Amynthas* sp. 3 | MH995899 |
| *Amynthas* sp. 4 | MH995900 |
| *Amynthas* sp. 5 | MH995901 |
| *Amynthas* sp. 6 | MH995902 |
| *Amynthas* sp. 7 | MH995903 |
| *Amynthas* sp. 8 | MH995904 |
| *Amynthas* sp. 9 | MH995905 |
| *Amynthas* sp. 10 | MH995906 |
| *Amynthas* sp. 11 | MH995907 |
| *Amynthas* sp. 12 | MH995908 |
| *Andiorrhinus* sp. 1 | MH995878 |
| *Andiorrhinus* sp. 2 | MH995879 |
| *Andiorrhinus* sp. 3 | MH995880 |
| *Aphelochaeta* sp. 1 | MH996356 |
| *Aphelochaeta* sp. 2 | MH996357 |
| *Aphelochaeta* sp. 3 | MH995881 |
| *Aphelochaeta* sp. 4 | MH995882 |
| *Aphelochaeta* sp. 5 | MH996358 |
| *Aphrodita japonica* 1 | MH995883 |
| *Aphrodita japonica* 2 | MH996359 |
| *Aphrodita japonica* 3 | MH995884 |
| *Aphrodita japonica* 4 | MH995885 |
| *Aphrodita japonica* 5 | MH996360 |
| *Aphrodita japonica* 6 | MH995886 |
| *Aphrodita japonica* 7 | MH995887 |
| *Arabella* sp. 1 | MH995888 |
| *Arabella* sp*.* 2 | MH995889 |
| *Arabella* sp*.* 3 | MH995890 |
| *Aricidea quadrilobata* 1 | MH995891 |
| *Aricidea quadrilobata* 2 | MH995892 |
| *Aricidea quadrilobata* 3 | MH995893 |
| *Aricidea quadrilobata* 4 | MH996361 |
| *Armandia* sp*.* 1 | MH995894 |
| *Armandia* sp*.* 2 | MH995895 |
| *Armandia* sp*.* 3 | MH995896 |
| *Auchenoplax crinita* 1 | MH995910 |
| *Auchenoplax crinita* 2 | MH995911 |
| *Auchenoplax crinita* 3 | MH995912 |
| *Auchenoplax crinita* 4 | MH995913 |
| *Aulodrilus japonicus* 1 | MH995914 |
| *Aulodrilus japonicus* 2 | MH995915 |
| *Aulodrilus japonicus* 3 | MH996363 |
| *Axiothella rubrocincta* 1 | MH996364 |
| *Axiothella rubrocincta* 2 | MH995916 |
| *Axiothella rubrocincta* 3 | MH995917 |
| *Axiothella rubrocincta* 4 | MH995918 |
| *Axiothella rubrocincta* 5 | MH995919 |
| *Axiothella rubrocincta* 6 | MH995920 |
| *Bathydrilus rohdei* | MH996365 |
| *Bispira pacifica* 1 | MH996261 |
| *Bispira pacifica* 2 | MH996262 |
| *Bispira pacifica* 3 | MH996263 |
| *Bispira pacifica* 4 | MH996264 |
| *Bispira pacifica* 5 | MH996265 |
| *Bispira pacifica* 6 | MH996266 |
| *Bispira pacifica* 7 | MH996267 |
| *Bothrioneurum vejdovskyanum* 1 | MH995921 |
| *Bothrioneurum vejdovskyanum* 2 | MH995922 |
| *Bothrioneurum vejdovskyanum* 3 | MH995923 |
| *Capilloventer* sp. 1 | MH996366 |
| *Capilloventer* sp. 2 | MH995924 |
| *Chaetozone* sp. 1 | MH995927 |
| *Chaetozone* sp. 2 | MH995928 |
| *Chaetozone* sp. 3 | MH995929 |
| *Chaetozone* sp. 4 | MH995930 |
| *Chaetozone* sp. 5 | MH996367 |
| *Chloeia pinnata* 1 | MH995931 |
| *Chloeia pinnata* 2 | MH995932 |
| *Chloeia pinnata* 3 | MH996368 |
| *Chloeia pinnata* 4 | MH995933 |
| *Chloeia pinnata* 5 | MH995934 |
| *Chloeia pinnata* 6 | MH995935 |
| *Chloeia pinnata* 7 | MH995936 |
| *Cirratulus spectabilis* 1 | MH995937 |
| *Cirratulus spectabilis* 2 | MH995938 |
| *Cirratulus spectabilis* 3 | MH995939 |
| *Clymenella torquata* 1 | MH995940 |
| *Clymenella torquata* 2 | MH995941 |
| *Clymenella torquata* 3 | MH995942 |
| *Clymenella torquata* 4 | MH995943 |
| *Clymenella torquata* 5 | MH995944 |
| *Clymenella torquata* 6 | MH995945 |
| *Clymenella torquata* 7 | MH996369 |
| *Clymenella torquata* 8 | MH996370 |
| *Cossura longocirrata* 1 | MH995946 |
| *Cossura longocirrata* 2 | MH995947 |
| *Cossura longocirrata* 3 | MH995948 |
| *Cossura longocirrata* 4 | MH995949 |
| *Cossura longocirrata* 5 | MH995950 |
| *Cossura longocirrata* 6 | MH995951 |
| *Cossura longocirrata* 7 | MH995952 |
| *Cossura longocirrata* 8 | MH995953 |
| *Cossura longocirrata* 9 | MH995954 |
| *Cossura longocirrata* 10 | MH995955 |
| *Cossura longocirrata* 11 | MH995956 |
| *Crucigera zygophora* | MH995957 |
| *Delaya leruthi* 1 | MH995958 |
| *Delaya leruthi* 2 | MH995959 |
| *Delaya leruthi* 3 | MH995960 |
| *Delaya leruthi* 4 | MH995961 |
| *Dichogaster* green tree worm 1 | MH995962 |
| *Dichogaster* green tree worm 2 | MH995963 |
| *Dichogaster* green tree worm 3 | MH995964 |
| *Dichogaster guadeloupensis* 1 | MH995965 |
| *Dichogaster guadeloupensis* 2 | MH995966 |
| *Dichogaster guadeloupensis* 3 | MH995967 |
| *Dichogaster saliens* | MH995968 |
| *Diplocardia* sp. 1 | MH995969 |
| *Diplocardia* sp. 2 | MH996371 |
| *Diplocardia* sp. 3 | MH995970 |
| *Diplocardia* sp. 4 | MH995971 |
| *Diplocardia* sp. 5 | MH995972 |
| *Diplocardia* sp. 6 | MH995973 |
| *Dodecaceria pulchra* 1 | MH995974 |
| *Dodecaceria pulchra* 2 | MH995975 |
| *Dodecaceria pulchra* 3 | MH995976 |
| *Dodecaceria pulchra* 4 | MH995977 |
| *Dodecaceria pulchra* 5 | MH995978 |
| *Dorydrilus michaelseni* 1 | MH995979 |
| *Dorydrilus michaelseni* 2 | MH995980 |
| *Dorydrilus michaelseni* 3 | MH995981 |
| *Drawida* sp. 1 | MH995982 |
| *Drawida* sp. 2 | MH995983 |
| *Drilocrius* sp*.* | MH995984 |
| Echiura gen. sp. green 1 | MH995985 |
| Echiura gen. sp. green 2 | MH995986 |
| *Eisenia* sp*.* 1 | MH995987 |
| *Eisenia* sp*.* 2 | MH995988 |
| *Eisenia* sp*.* 3 | MH995989 |
| *Eisenia* sp*.* 4 | MH995990 |
| *Eisenia* sp*.* 5 | MH995991 |
| *Enchytraeus albidus* | MH995992 |
| *Erpobdella octoculata* 1 | MH995993 |
| *Erpobdella octoculata* 2 | MH995994 |
| *Erpobdella octoculata* 3 | MH995995 |
| *Eunice norvegica* 1 | MH995996 |
| *Eunice norvegica* 2 | MH995997 |
| *Eunice norvegica* 3 | MH995998 |
| *Eunice pennata* 1 | MH995999 |
| *Eunice pennata* 2 | MH996000 |
| *Eunice pennata* 3 | MH996001 |
| *Flabegraviera mundata* | MH996002 |
| *Galathowenia oculata* 1 | MH996003 |
| *Galathowenia oculata* 2 | MH996004 |
| *Galathowenia oculata* 3 | MH996005 |
| *Galathowenia oculata* 4 | MH996006 |
| *Galeolaria caespitosa* 1 | MH996007 |
| *Galeolaria caespitosa* 2 | MH996008 |
| *Gatesona chaetophora* | MH996009 |
| *Geogenia benhami* 1 | MH996010 |
| *Geogenia benhami* 2 | MH996011 |
| *Glossodrilus* sp*.* | MH996012 |
| *Glycera dibranchiata* 1 | MH996013 |
| *Glycera dibranchiata* 2 | MH996014 |
| *Glycera dibranchiata* 3 | MH996015 |
| *Glycera dibranchiata* 4 | MH996016 |
| *Glyptonotobdella antarctica* 1 | MH996017 |
| *Glyptonotobdella antarctica* 2 | MH996018 |
| *Glyptonotobdella antarctica* 3 | MH996019 |
| *Goniada brunnea* 1 | MH996020 |
| *Goniada brunnea* 2 | MH996021 |
| *Grania* sp*.* 1 | MH996022 |
| *Grania* sp*.* 2 | MH996023 |
| *Grania* sp*.* 3 | MH996024 |
| *Guaranidrilus* sp*.* 1 | MH996025 |
| *Guaranidrilus* sp*.* 2 | MH996372 |
| *Halosydna brevisetosa* 1 | MH996373 |
| *Halosydna brevisetosa* 2 | MH996026 |
| *Halosydna brevisetosa* 3 | MH996027 |
| *Halosydna brevisetosa* 4 | MH996028 |
| *Halosydna brevisetosa* 5 | MH996029 |
| *Halosydna brevisetosa* 6 | MH996030 |
| Haplotaxidae gen. sp. 1 - 1 | MH996031 |
| Haplotaxidae gen. sp. 1 - 2 | MH996032 |
| *Haplotaxis gordioides* | MH996033 |
| *Haplotaxis* sp. | MH996034 |
| *Hemigastrodrilus monicae* | MH996035 |
| *Hermodice carunculata* 1 | MH996039 |
| *Hermodice carunculata* 2 | MH996040 |
| *Hermodice carunculata* 3 | MH996041 |
| *Heronidrilus* sp. 1 | MH996042 |
| *Heronidrilus* sp. 2 | MH996043 |
| *Heronidrilus* sp. 3 | MH996044 |
| *Heronidrilus* sp. 4 | MH996045 |
| *Heronidrilus* sp. 5 | MH996046 |
| *Hesionides* sp*.* 1 | MH996047 |
| *Hesionides* sp*.* 2 | MH996048 |
| *Hesionides* sp*.* 3 | MH996049 |
| *Hesionides* sp*.* 4 | MH996050 |
| *Heterodrilus* sp. 1 - 1 | MH996376 |
| *Heterodrilus* sp. 1 - 2 | MH996051 |
| *Heterodrilus* sp. 1 - 3 | MH996052 |
| *Idanthyrsus* sp. | MH996053 |
| *Kincaidiana* sp. 1 | MH996054 |
| *Kincaidiana* sp. 2 | MH996055 |
| *Kincaidiana* sp. 3 | MH996056 |
| *Kincaidiana* sp. 4 | MH996057 |
| *Komarekiona eatoni* | MH996058 |
| *Lamellibrachia luymesi* 1 | MH996063 |
| *Lamellibrachia luymesi* 2 | MH996064 |
| *Lamellibrachia luymesi* 3 | MH996065 |
| *Lamellibrachia luymesi* 4 | MH996378 |
| *Lamellibrachia luymesi* 5 | MH996066 |
| *Lamellibrachia luymesi* 6 | MH996067 |
| *Lamellibrachia luymesi* 7 | MH996068 |
| *Lamellibrachia luymesi* 8 | MH996069 |
| *Lamellibrachia luymesi* 9 | MH996070 |
| *Lamellibrachia luymesi* 10 | MH996379 |
| *Laonice* sp. | MH996071 |
| *Leitoscoloplos robustus* 1 | MH996072 |
| *Leitoscoloplos robustus* 2 | MH996073 |
| *Leitoscoloplos robustus* 3 | MH996380 |
| *Leitoscoloplos robustus* 4 | MH996074 |
| *Lepidonotus semitectus* 1 | MH996075 |
| *Lepidonotus semitectus* 2 | MH996076 |
| *Lepidonotus semitectus* 3 | MH996077 |
| *Limnodriloides* sp*.* 1 | MH996081 |
| *Limnodriloides* sp*.* 2 | MH996082 |
| *Limnodriloides* sp*.* 3 | MH996083 |
| *Limnodriloides* sp*.* 4 | MH996084 |
| *Limnodriloides* sp*.* 5 | MH996085 |
| *Limnodriloides* sp*.* 6 | MH996086 |
| *Limnodriloides* sp*.* 7 | MH996087 |
| *Lumbriculus variegatus* 1 | MH996381 |
| *Lumbriculus variegatus* 2 | MH996088 |
| *Lumbriculus variegatus* 3 | MH996089 |
| *Lumbriculus variegatus* 4 | MH996090 |
| *Lumbrineris crassicephala* 1 | MH996091 |
| *Lumbrineris crassicephala* 2 | MH996092 |
| *Lumbrineris crassicephala* 3 | MH996093 |
| *Lumbrineris crassicephala* 4 | MH996094 |
| *Lumbrineris crassicephala* 5 | MH996095 |
| *Lumbrineris crassicephala* 6 | MH996096 |
| *Lumbrineris perkinsi* 1 | MH996097 |
| *Lumbrineris perkinsi* 2 | MH996098 |
| *Lumbrineris perkinsi* 3 | MH996099 |
| *Lutodrilus* sp. 1 | MH996100 |
| *Lutodrilus* sp. 2 | MH996101 |
| *Lutodrilus* sp. 3 | MH996102 |
| *Lutodrilus* sp. 4 | MH996103 |
| *Macrochaeta* sp. | MH996382 |
| *Magelona berkeleyi* 1 | MH996104 |
| *Magelona berkeleyi* 2 | MH996105 |
| *Magelona berkeleyi* 3 | MH996106 |
| *Magelona berkeleyi* 4 | MH996107 |
| *Marphysa sanguinea* 1 | MH996108 |
| *Marphysa sanguinea* 2 | MH996383 |
| *Marphysa sanguinea* 3 | MH996109 |
| *Marphysa sanguinea* 4 | MH996110 |
| *Melinna maculata* 1 | MH996111 |
| *Melinna maculata* 2 | MH996112 |
| *Melinna maculata* 3 | MH996384 |
| *Melinna maculata* 4 | MH996113 |
| *Melinna maculata* 5 | MH996385 |
| *Mesenchytraeus pedatus* 1 | MH996114 |
| *Mesenchytraeus pedatus* 2 | MH996115 |
| *Mesenchytraeus solifugus* DARK | MH996116 |
| Microchaetidae gen. sp. 1 - 1 | MH996117 |
| Microchaetidae gen. sp. 1 - 2 | MH996118 |
| *Microchaetus* sp. | MH996119 |
| *Microphthalmus similis* | MH996120 |
| *Myxicola infundibulum* 1 | MH996121 |
| *Myxicola infundibulum* 2 | MH996122 |
| *Myxicola infundibulum* 3 | MH996123 |
| *Myxicola infundibulum* 4 | MH996124 |
| *Myxicola infundibulum* 5 | MH996125 |
| *Myxicola infundibulum* 6 | MH996126 |
| *Naineris laevigata* 1 | MH996127 |
| *Naineris laevigata* 2 | MH996128 |
| *Naineris laevigata* 3 | MH996129 |
| *Naineris laevigata* 4 | MH996130 |
| *Naineris laevigata* 5 | MH996131 |
| *Naineris laevigata* 6 | MH996132 |
| *Naineris laevigata* 7 | MH996133 |
| *Naineris laevigata* 8 | MH996134 |
| *Naineris laevigata* 9 | MH996135 |
| *Naineris laevigata* 10 | MH996136 |
| *Naineris laevigata* 11 | MH996137 |
| *Neosabellaria cementarium* 1 | MH996138 |
| *Neosabellaria cementarium* 2 | MH996139 |
| *Neosabellaria cementarium* 3 | MH996140 |
| *Nephtys incisa* 1 | MH996141 |
| *Nephtys incisa* 2 | MH996142 |
| *Nephtys incisa* 3 | MH996386 |
| *Nephtys incisa* 4 | MH996387 |
| *Nicolea macrobranchia* | MH996148 |
| *Nicomache venticola* 1 | MH996388 |
| *Nicomache venticola* 2 | MH996149 |
| *Nicomache venticola* 3 | MH996150 |
| *Ninoe nigripes* 1 | MH996151 |
| *Ninoe nigripes* 2 | MH996152 |
| *Ninoe nigripes* 3 | MH996153 |
| *Ninoe nigripes* 4 | MH996154 |
| *Ninoe nigripes* 5 | MH996389 |
| *Odontosyllis gibba* 1 | MH996390 |
| *Odontosyllis gibba* 2 | MH996157 |
| *Oenone fulgida* 1 | MH996391 |
| *Oenone fulgida* 2 | MH996158 |
| *Oenone fulgida* 3 | MH996159 |
| *Oenone fulgida* 4 | MH996160 |
| *Olavius (Coralliodriloides) loisae* 1 | MH996162 |
| *Olavius (Coralliodriloides) loisae* 2 | MH996163 |
| *Olavius albidus* 1 | MH996392 |
| *Olavius albidus* 2 | MH996161 |
| Oligochaeta gen. sp. (unidentified Crassiclitellata - Place Kabary 2) - 1 | MH996214 |
| Oligochaeta gen. sp. (unidentified Crassiclitellata - Place Kabary 2) - 2 | MH996215 |
| Oligochaeta gen. sp. (unidentified Crassiclitellata - Place Kabary 2) - 3 | MH996216 |
| Oligochaeta gen. sp. (unidentified Crassiclitellata - Place Kabary 2) - 4 | MH996217 |
| Oligochaeta gen. sp. (unidentified Crassiclitellata - Place Kabary 2) - 5 | MH996218 |
| *Ophelina acuminata* 1 | MH996164 |
| *Ophelina acuminata* 2 | MH996165 |
| *Ophelina acuminata* 3 | MH996166 |
| *Ophelina acuminata* 4 | MH996393 |
| *Ophelina acuminata* 5 | MH996167 |
| *Osedax mucofloris* 1 | MH996168 |
| *Osedax mucofloris* 2 | MH996394 |
| *Owenia fusiformis* 1 | MH996395 |
| *Owenia fusiformis* 2 | MH996169 |
| *Owenia fusiformis* 3 | MH996170 |
| *Palola* sp*.* 1 | MH996171 |
| *Palola* sp*.* 2 | MH996172 |
| *Palola* sp*.* 3 | MH996173 |
| *Palola* sp*.* 4 | MH996174 |
| *Palola* sp*.* 5 | MH996175 |
| *Parachilota* sp. 1 | MH996176 |
| *Parachilota* sp. 2 | MH996177 |
| *Parachilota* sp. 3 | MH996178 |
| *Paralvinella palmiformis* 1 | MH996396 |
| *Paralvinella palmiformis* 2 | MH996397 |
| *Paramphinome jeffreysii* 1 | MH996179 |
| *Paramphinome jeffreysii* 2 | MH996180 |
| *Paramphinome jeffreysii* 3 | MH996181 |
| *Paramphinome jeffreysii* 4 | MH996398 |
| *Paramphinome jeffreysii* 5 | MH996399 |
| *Paranais* sp*.* 1 | MH996182 |
| *Paranais* sp*.* 2 | MH996400 |
| *Pectinaria gouldii* 1 | MH996187 |
| *Pectinaria gouldii* 2 | MH996188 |
| *Pectinaria gouldii* 3 | MH996189 |
| *Pectinaria gouldii* 4 | MH996190 |
| *Pectinaria gouldii* 5 | MH996191 |
| *Pectinaria gouldii* 6 | MH996192 |
| *Pectinaria gouldii* 7 | MH996193 |
| *Perinereis* sp. 1 | MH996203 |
| *Perinereis* sp. 2 | MH996204 |
| *Phagodrilus* sp*.* 1 | MH996205 |
| *Phagodrilus* sp*.* 2 | MH996206 |
| *Pherecardia striata* 1 | MH996207 |
| *Pherecardia striata* 2 | MH996208 |
| *Pherecardia striata* 3 | MH996209 |
| *Pherusa plumosa* | MH996403 |
| Phreodrilidae gen. sp. 1 - 1 | MH996212 |
| Phreodrilidae gen. sp. 1 - 2 | MH996213 |
| *Poeobius meseres* 1 | MH996197 |
| *Poeobius meseres* 2 | MH996198 |
| *Poeobius meseres* 3 | MH996199 |
| *Poeobius meseres* 4 | MH996200 |
| *Poeobius meseres* 5 | MH996201 |
| *Poeobius meseres* 6 | MH996202 |
| *Pontodrilus litoralis* 1 | MH996224 |
| *Pontodrilus litoralis* 2 | MH996404 |
| *Pontodrilus litoralis* 3 | MH996225 |
| *Pontodrilus litoralis* 4 | MH996226 |
| *Pontodrilus litoralis* 5 | MH996227 |
| *Pontodrilus litoralis* 6 | MH996228 |
| *Praxillella pacifica* 1 | MH996229 |
| *Praxillella pacifica* 2 | MH996230 |
| *Praxillella pacifica* 3 | MH996231 |
| *Praxillella pacifica* 4 | MH996232 |
| *Praxillella pacifica* 5 | MH996233 |
| *Prionospio dubia* 1 | MH996234 |
| *Prionospio dubia* 2 | MH996235 |
| *Prionospio dubia* 3 | MH996236 |
| *Prionospio dubia* 4 | MH996237 |
| *Prionospio dubia* 5 | MH996238 |
| *Prionospio dubia* 6 | MH996239 |
| *Propappus volki* 1 | MH996243 |
| *Propappus volki* 2 | MH996244 |
| *Propappus volki* 3 | MH996245 |
| *Proscoloplos cygnochaetus* 1 | MH996246 |
| *Proscoloplos cygnochaetus* 2 | MH996247 |
| *Proscoloplos cygnochaetus* 3 | MH996248 |
| *Proscoloplos cygnochaetus* 4 | MH996406 |
| *Pseudonereis variegata* | MH996407 |
| *Randiella* sp*.* 1 | MH996249 |
| *Randiella* sp*.* 2 | MH996250 |
| *Randiella* sp*.* 3 | MH996251 |
| *Randiella* sp*.* 4 | MH996252 |
| *Rhinodrilus priollii* 1 | MH996253 |
| *Rhinodrilus priollii* 2 | MH996254 |
| *Rhinodrilus priollii* 3 | MH996255 |
| *Rhyacodrilus falciformis* 1 | MH996256 |
| *Rhyacodrilus falciformis* 2 | MH996257 |
| *Rhyacodrilus falciformis* 3 | MH996258 |
| *Sabaco elongatus* 1 | MH996259 |
| *Sabaco elongatus* 2 | MH996260 |
| *Scalibregma inflatum* 1 | MH996268 |
| *Scalibregma inflatum* 2 | MH996269 |
| *Scalibregma inflatum* 3 | MH996270 |
| *Scalibregma inflatum* 4 | MH996271 |
| *Scalibregma inflatum* 5 | MH996272 |
| *Scalibregma inflatum* 6 | MH996408 |
| *Scalibregma inflatum* 7 | MH996273 |
| *Scalibregma inflatum* 8 | MH996274 |
| *Sclerolinum brattstromi* 1 | MH996275 |
| *Sclerolinum brattstromi* 2 | MH996276 |
| *Sclerolinum brattstromi* 3 | MH996277 |
| *Sclerolinum brattstromi* 4 | MH996278 |
| *Sclerolinum brattstromi* 5 | MH996409 |
| *Sclerolinum brattstromi* 6 | MH996279 |
| *Sclerolinum brattstromi* 7 | MH996280 |
| *Sclerolinum brattstromi* 8 | MH996410 |
| *Scolelepis squamata* | MH996411 |
| *Serpula vermicularis* 1 | MH996283 |
| *Serpula vermicularis* 2 | MH996284 |
| *Serpula vermicularis* 3 | MH996285 |
| *Serpula vermicularis* 4 | MH996286 |
| *Serpula vermicularis* 5 | MH996287 |
| *Siboglinum ekmani* 1 | MH996288 |
| *Siboglinum ekmani* 2 | MH996289 |
| *Siboglinum ekmani* 3 | MH996290 |
| *Siboglinum ekmani* 4 | MH996291 |
| *Siboglinum ekmani* 5 | MH996292 |
| *Siboglinum ekmani* 6 | MH996293 |
| *Siboglinum ekmani* 7 | MH996412 |
| *Sparganophilus* sp. 1 | MH996413 |
| *Sparganophilus* sp. 2 | MH996414 |
| *Sparganophilus* sp. 3 | MH996294 |
| *Sparganophilus* sp. 4 | MH996415 |
| *Spirobranchus kraussii* 1 | MH996219 |
| *Spirobranchus kraussii* 2 | MH996220 |
| *Spirobranchus kraussii* 3 | MH996221 |
| *Spirobranchus kraussii* 4 | MH996222 |
| *Spirobranchus kraussii* 5 | MH996223 |
| *Sternaspis scutata* 1 | MH996419 |
| *Sternaspis scutata* 2 | MH996303 |
| *Sternaspis scutata* 3 | MH996304 |
| *Sternaspis scutata* 4 | MH996420 |
| *Sternaspis scutata* 5 | MH996305 |
| *Sternaspis scutata* 6 | MH996306 |
| *Sternaspis scutata* 7 | MH996307 |
| *Sternaspis scutata* 8 | MH996421 |
| *Sternaspis scutata* 9 | MH996308 |
| *Sternaspis* sp*.* 1 | MH996299 |
| *Sternaspis* sp*.* 2 | MH996300 |
| *Sternaspis* sp*.* 3 | MH996417 |
| *Sternaspis* sp*.* 4 | MH996418 |
| *Sternaspis* sp*.* 5 | MH996301 |
| *Sternaspis* sp*.* 6 | MH996302 |
| *Stylodrilus heringianus* 1 | MH996309 |
| *Stylodrilus heringianus* 2 | MH996310 |
| *Stylodrilus heringianus* 3 | MH996311 |
| *Stylodrilus heringianus* 4 | MH996312 |
| *Syllis* cf. *hyalina* 1 | MH996313 |
| *Syllis* cf. *hyalina* 2 | MH996314 |
| *Syllis* cf. *hyalina* 3 | MH996315 |
| *Syllis* cf. *hyalina* 4 | MH996316 |
| *Syllis* cf. *hyalina* 5 | MH996317 |
| *Syllis* cf. *hyalina* 6 | MH996318 |
| *Syllis* cf. *hyalina* 7 | MH996319 |
| *Syllis* cf. *hyalina* 8 | MH996320 |
| *Syllis* cf. *hyalina* 9 | MH996321 |
| *Terebellides stroemii* 1 | MH996322 |
| *Terebellides stroemii* 2 | MH996323 |
| *Terebellides stroemii* 3 | MH996324 |
| *Terebellides stroemii* 4 | MH996325 |
| *Terebellides stroemii* 5 | MH996326 |
| *Terebellides stroemii* 6 | MH996422 |
| *Terebellides stroemii* 7 | MH996327 |
| *Terebellides stroemii* 8 | MH996328 |
| *Terebellides stroemii* 9 | MH996329 |
| *Terebellides stroemii* 10 | MH996330 |
| *Terebellides stroemii* 11 | MH996331 |
| *Terebellides stroemii* 12 | MH996332 |
| *Thalassodrilides* sp*.* 1 | MH996333 |
| *Thalassodrilides* sp*.* 2 | MH996334 |
| *Thalassodrilides* sp*.* 3 | MH996335 |
| *Thalassodrilides* sp*.* 4 | MH996336 |
| *Thalassodrilides* sp*.* 5 | MH996423 |
| *Tharyx kirkegaardi* 1 | MH996337 |
| *Tharyx kirkegaardi* 2 | MH996424 |
| *Tharyx kirkegaardi* 3 | MH996338 |
| *Thelepus crispus* | MH996339 |
| *Thysanocardia nigra* 1 | MH996340 |
| *Thysanocardia nigra* 2 | MH996341 |
| *Thysanocardia nigra* 3 | MH996342 |
| *Timarete punctata* 1 | MH996343 |
| *Timarete punctata* 2 | MH996344 |
| *Tomopteris* sp. 1 | MH996345 |
| *Tomopteris* sp. 2 | MH996346 |
| *Tomopteris* sp. 3 | MH996347 |
| *Travisia brevis* 1 | MH996352 |
| *Travisia brevis* 2 | MH996425 |
| *Travisia brevis* 3 | MH996353 |
| *Troglodrilus jugeti* | MH996354 |
| *Vignysa popi* | MH996355 |
| **Brachiopoda** |  |
| *Hemithiris psittacea* 1 | MH996374 |
| *Hemithiris psittacea* 2 | MH996375 |
| *Hemithiris psittacea* 3 | MH996036 |
| *Hemithiris psittacea* 4 | MH996037 |
| *Hemithiris psittacea* 5 | MH996038 |
| *Novocrania anomala* 1 | MH996155 |
| *Novocrania anomala* 2 | MH996156 |
| **Phoronida** |  |
| *Phoronis psammophila* 1 | MH996210 |
| *Phoronis psammophila* 2 | MH996211 |
| **Mollusca** |  |
| *Hypomenia* sp*.* | MH996377 |
| *Kruppomenia borealis* 1 | MH996059 |
| *Kruppomenia borealis* 2 | MH996060 |
| *Leptochiton rugatus* 1 | MH996078 |
| *Leptochiton rugatus* 2 | MH996079 |
| *Leptochiton rugatus* 3 | MH996080 |
| *Spathoderma clenchi* | MH996295 |
| **Nemertea** |  |
| *Paranemertes peregrina* 1 | MH996183 |
| *Paranemertes peregrina* 2 | MH996401 |
| *Paranemertes peregrina* 3 | MH996184 |
| *Paranemertes peregrina* 4 | MH996402 |
| *Paranemertes peregrina* 5 | MH996185 |
| *Paranemertes peregrina* 6 | MH996186 |
| **Bryozoa** |  |
| *Pectinatella magnifica* 1 | MH996194 |
| *Pectinatella magnifica* 2 | MH996195 |
| *Pectinatella magnifica* 3 | MH996196 |
| **Platyhelminthes** |  |
| *Selachohemecus olsoni* 1 | MH996281 |
| *Selachohemecus olsoni* 2 | MH996282 |
| **Priapulida** |  |
| *Priapulus* sp*.* 1 | MH996405 |
| *Priapulus* sp*.* 2 | MH996240 |
| *Priapulus* sp*.* 3 | MH996241 |
| *Priapulus* sp*.* 4 | MH996242 |
